# Proanthocyanidins-loaded complex coacervates-based drug delivery attenuates oral squamous cell carcinoma cells metastatic potential through down-regulating the Akt signaling pathway

**DOI:** 10.3389/fonc.2022.1001126

**Published:** 2022-10-18

**Authors:** Ju-Fang Liu, Yinshen Wee, Shen-Dean Luo, Shwu-Fen Chang, Shihai Jia, Sheng-Wei Feng, Huei-Mei Huang, Jiann-Her Lin, Ching-Shuen Wang

**Affiliations:** ^1^ School of Oral Hygiene, College of Oral Medicine, Taipei Medical University, Taipei, Taiwan; ^2^ Department of Pathology, University of Utah, Salt Lake City, UT, United States; ^3^ Department of Otolaryngology, Kaohsiung Chang Gung Memorial Hospital and Chang Gung University College of Medicine, Kaohsiung, Taiwan; ^4^ Graduate Institute of Medical Sciences, College of Medicine, Taipei Medical University, Taipei, Taiwan; ^5^ Department of Neurobiology, University of Utah, Salt Lake City, UT, United States; ^6^ School of Dentistry, College of Oral Medicine, Taipei Medical University, Taipei, Taiwan; ^7^ Department of Neurosurgery, Taipei Medical University Hospital, Taipei, Taiwan

**Keywords:** complex coacervates, novel drug carrier, grape seed proanthocyanidins, chemopreventive agent, oral squamous cell carcinoma, protein kinase B, matrix metalloproteinases

## Abstract

Oral cancer, constituted up to 90% by squamous cell carcinomas, is a significant health burden globally. Grape seed proanthocyanidins (PA) have been suggested as a potential chemopreventive agent for oral cancer. However, their efficacy can be restricted due to the low bioavailability and bioaccessibility. Inspired by sandcastle worm adhesive, we adapted the concept of complex coacervation to generate a new type of drug delivery platform. Complex coacervates are a dense liquid phase formed by the associative separation of a mixture of oppositely charged polyelectrolytes, can serve as a drug delivery platform to protect labile cargo. In this study, we developed a complex coacervates-based delivery of PA. The release kinetics was measured, and anticancer effects were determined in two human tongue squamous cell carcinoma cell lines. The results showed that complex coacervate successfully formed and able to encapsulate PA. Additionally, PA were steadily released from the system in a pH-dependent manner. The drug delivery system could significantly inhibit the cell proliferation, migration, and invasion of cancer cells. Moreover, it could markedly reduce the expression of certain matrix metalloproteinases (MMP-2, 9, and 13) crucial to metastatic processes. We also found that suppression of protein kinase B (Akt) pathway might be the underlying mechanism for these anticancer activities. Taken together, complex coacervates-based delivery of PA can act as an effective anticancer approach for oral cancer therapy.

## Introduction

Oral cancer is a prevalent malignancy in the head and neck region with aggressive nature and poor prognosis (1). It is a significant global health issue, with more than 377,000 new cases and 177,000 deaths worldwide in 2020. The global estimation also suggests that oral cancer incidence and mortality will further increase in coming decades (2). Despite the advancements in oral cancer treatment, prognosis and survival rate for patients have not been significantly improved. Treatment failure mainly depends on disease stages and therapeutic restrictions (3, 4). Therefore, the research and development of novel effective approaches are required for preventing and treating this fatal disease.

Natural products are compounds derived from natural sources, which are beneficial for human health (5). Flavonoids refer to as an important class of natural products having polyphenolic structures, which are abundantly found in fruits, vegetables, and beverages (6). Grape seeds are rich sources of proanthocyanidins (PA) that are among the most common subgroup of flavonoids. Dietary consumption of PA is considered safe and beneficial for human health. The benefits of PA are mainly attributed to their anti-oxidant, antimicrobial, and anti-inflammatory properties (7). Furthermore, grape seed PA are now appreciated for their multiple anticancer activities such as anti-proliferative, pro-apoptotic, anti-angiogenic, and anti-metastatic effects (7–9). Past studies demonstrated the chemopreventive effects of PA to prevent the development and progression of oral cancer both *in vitro* and *in vivo* (10, 11). However, their efficacy in clinical practice can be restricted due to the low bioavailability referring to as the proportion of a compound that is ingested, absorbed, digested, and reached the systemic circulation. In addition, due to their chemical nature to promptly bind to fiber, sugar, and protein molecules, most PA are insoluble, thereby compromising their bioaccessibility (7). Therefore, the improvement of technologies to enhance the bioavailability and bioaccessibility of PA would be advantageous to optimize their anticancer effects.

Encapsulation is generally deployed in drug delivery systems to form a shell in order to protect a particular agent and prevent it from leaching out before reaching the target site (12). Sandcastle worms living along the coast build their shells under seawater by secreting a multicomponent, self-initiating, and rapidly-set adhesive (13, 14). The phenomenon of complex coacervation, which is an associative separation of oppositely charged polyelectrolytes into liquid-liquid phases, plays an important role in the formation of the adhesive of sandcastle worms (15). With the advancements of drug delivery technology, the use of complex coacervates as a drug delivery platform has been emerged for their high loading capacity and self-assembly in aqueous medium (16, 17). Once encapsulated within the coacervate phase, a particular agent can be protected from the surrounding environment to preserve its integrity and bioactivity, subsequently increasing the bioavailability and bioaccessibility. Moreover, unlike other common vehicles such as hydrogels and microparticles, coacervates can form quickly and do not require organic solvents which can adversely affect the drug retention and bioactivity (16).

Encouraged by the promising concept of complex coacervate structures, we developed a complex coacervates-based drug carrier (CCDC) for delivering grape seed PA with a hope to enhance the bioavailability and biodistribution of PA, thereby improving their performance. Aside from the advantage that encapsulation can protect a cargo molecule during the administration, two critical aspects of a drug delivery system include the sufficient release from the carrier and the subsequent induction of biological effects in the target site (18). Therefore, in this study, the kinetic release profile of CCDC-PA system as well as its inhibitory effects on cancer progression including cell proliferation, cell migration, cell invasion, and matrix metalloproteinases (MMPs) expression were investigated. In addition, literature has indicated that anticancer activities of grape seed PA mostly rely on their orchestration of various signaling pathways such as phosphatidylinositol 3-kinase/protein kinase B (PI3K/Akt) and mitogen-activated protein kinases (MAPKs) (8). Hence, these pathways were also studied in this research to reveal the possible underlying mechanisms by which CCDC-PA system functioned. Since the majority of oral cancer cases is comprised of oral squamous cell carcinoma (OSCC) with the tongue is the most common site (19), SCC4 and HSC-3 cell lines were employed in this study. The former cell line is derived from human tongue squamous cell carcinoma at T3N0M0 stage, whereas the latter is established from tumors of metastatic lymph nodes originated in human tongue squamous cell carcinoma.

## Material and methods

### Reagents

Oligochitosan (Och), Sodium hexametaphosphate (P6), and Proanthocyanidins (PA) were purchased from Sigma, Taiwan. All solutions were prepared in ultrapure water and undissolved debris were removed by 0.22 micron filters and stored at 4°C before use.

### Preparation of complex coacervates-based drug carrier

Complex coacervates-based drug carrier was prepared by mixing Och (10 mg/mL) and P6 (10 mg/mL) at different ratio. Briefly, Och and P6 were dissolved in ultrapure water at various pH from 2.0 to 8.0 with 1M HCl or 1N NaOH solution. The Och and P6 solutions were mixed intensively at different volume ratios ranging from 5:1 to 1:5 of Och/P6. Complex coacervates formed and the condensed liquid phase was centrifuged at 1,500 rpm at room temperature (RT) for 5 min. The yields of complex coacervates drug carrier (CCDC) at each condition were calculated by the equation (1):

Equation (1)


$$\%\;yield=\left[(CCDC_{0}-CCDC_{i})/CCDC_{0}\right]\times 100$$

where CCDC_0_ was the total powder weight of Och and P6 used to prepare the CCDC solution, and CCDC_i_ was the weight of the freeze-dried CCDC. The formation of CCDC was then visualized by bright-field microscopy (Leica, Germany) and fluorescence microscopy (Olympus, Japan).

### Procedure of PA encapsulation

Och was premixed with PA at various concentrations ranging from 20 mM to 1.25 mM. Then P6 was added to the premixed solutions to form the CCDC-PA system at different stoichiometric ratios (1:20, 1:10, 1:5, 1:2, and 1:1). After 30 min for settling down at RT, the supernatant was measured by using UV-vis spectrometry since the absorbance at 230 nm is specific for PA and it is not overlapped with that of Och at 274 nm. Determinations were performed in triplicate.

The encapsulation efficiency of each ratio was calculated using the Equation (2):

Equation (2)


$$Encapsulation\;efficiency=(UV_{0}/UV_{i})\times 100$$

where UV_0_ was the UV absorbance in the supernatant at the specific wavelength, and UV_i_ was the reference UV absorbance at the standard concentration. The concentration of PA can be accurately and quantified by linear regression method shown in **Supplemental Results**.

### Determination of release kinetics of PA from CCDC-PA

Kinetic release experiments of PA from CCDC-PA were carried out in different buffered pH solutions. CCDC-PA at various ratios was prepared as described above. The release kinetics of PA was determined by UV-vis spectrometry at the wavelength of 230 nm. Briefly, supernatants were sampled and replaced with 1 mL of fresh buffered pH solutions after 1, 2, 4, 8, 12, and 24 h. The replacement was then done every 24 h for 30 days. The corresponding cumulative percentage of released PA was determined using a standard calibration curve covering the range of the assay.

### Cell culture

SCC4 and HSC-3 cell lines were investigated throughout this study as representatives of human oral squamous cell carcinomas. SCC4 cell line was purchased from Bioresource Collection and Research Center (BCRC, Hsinchu, Taiwan). SCC4 cells were cultured in Dulbecco’s modified Eagle’s medium (DMEM)/F12 supplemented with 10% fetal bovine serum (FBS), 2 mM glutamine, 0.4 μg/ml hydrocortisone, 100 U/ml penicillin, and 100 μg/ml of streptomycin at 37°C in a humidified atmosphere of 5% (v/v) CO_2_ in air. HSC-3 cell line was purchased from Merck (Darmstadt, Hesse, Germany). HSC-3 cells were cultured in DMEM supplemented with 10% FBS at 37°C and 5% CO_2_. Cells were then seeded into 24-well plates one day before various doses of drug treatments (CCDC-PA, and SC78). After treatments, cells were then analyzed with the following techniques.

### Immunofluorescence microscopy

HSC-3 and SCC4 cells (5 × 10^3^ cells/well) were seeded on glass coverslips and treated with designed conditions. After rinsing once with PBS, the cells on the slice were fixed in 3.7% paraformaldehyde at RT for 15 min. Next, they were washed three times with PBS to remove the residuals of the fixation solution, and then 4% BSA was used for blocking for 15 min. The cells were incubated with anti-human CREB (1:100) at RT for 1 h. After washing twice with PBS, they were further incubated with FITC-conjugated goat anti-rabbit IgG for 1 h. Images were visualized under a fluorescence microscope (Zeiss, Axiovert 200 M).

### Cell proliferation assay

HSC-3 and SCC4 cells (8 × 10^3^ cells/well) were seeded on a 96-well plates and treated with designed conditions. After 24 h or 48 h, 10 μL of CCK-8 solution was added into every well, and the plates were incubated for 3 h to 6 h. The results were read on a microplate reader at the absorbance of 450 nm.

### Wound healing assay

HSC-3 and SCC4 cells (1 × 10^5^ cells/well) were seeded on 12-well plates. The confluent monolayer of culture was scratched with a fine pipette tip, and CCDC-PA at various ratios was added into the plates for 24 h. Following the treatments, cell migration was visualized under a microscope and the rate of wound closure was quantified.

### Cell migration assay

The Transwell inserts (8-μm pore size; Costar, NY, USA) in 24-well dishes were used for cell migration assay. HSC-3 and SCC4 cells (3 × 10^4^ cells/well) were treated with CCDC-PA at various ratios for 90 min and further incubated for 24 h. The cells were then seeded in the upper Transwell chamber, and 300 μL of culture medium was added into the lower chamber. After 24 h, the cells were fixed in 3.7% formaldehyde for 30 min and stained with 0.05% crystal violet for 60 min. Each chamber was washed with DDW after removing the cells on the top of the filter by using cotton-tipped swabs. The cells remained on the bottom of the filter were then examined and counted under a microscope.

### RNA extraction and quantitative real-time PCR

Total RNAs were isolated from HSC-3 and SCC4 cells treated with CCDC-PA at various ratios by using Total RNA preparation kits (easy-Blue Total RNA Extraction kit, iNtRON Biotechnology, Seongnam, Korea) following the manufacturer’s protocol. The RNA was reversely transcribed to cDNA by reverse transcriptase (Invitrogen, Carlsbad, CA, USA). Quantitative real-time PCR (qPCR) was used to determine the mRNA levels of target genes by running on the StepOnePlus machine (Applied Biosystems, Foster City, CA, USA). The SYBR Green fluorescence probe system (KAPA Biosystems, Woburn, MA, USA) was used for determining the threshold cycle (C_T_) of target genes. Primers of human MMP-2, MMP-9, MMP-12, MMP-13, and glyceraldehyde 3-phosphate dehydrogenase (GAPDH) were purchased from Sigma-Aldrich. The expression levels of target genes were normalized to GAPDH levels. The formula of level ratio of 2^−ΔΔCt^, where ΔΔCt = (Ct _target_−Ct _GADPH_)_Sample_−(Ct _target_−Ct _GADPH_)_Control_, was used for calculation.

### Immunoblotting assay

Total cell lysates were collected from HSC-3 and SCC4 cells treated with CCDC-PA at various ratios by using RIPA lysis buffer for immunoblotting assay. Briefly, equal amounts of proteins were separated by SDS-polyacrylamide gel electrophoresis and transferred to polyvinyl difluoride (PVDF) membranes. Blots were blocked with 5% non-fat milk at RT for 1 h, followed by overnight incubation at 4°C with specific primary antibodies at 1:1000 dilutions: ACTIN (Catalog No.: SI-A5441, Sigma); MMP2 (Catalog No.: 104577, Genetex), MMP9 (Catalog No.: 100458, Genetex), MMP12 (Catalog No.: 102928, Genetex), MMP13 (Catalog No.: 69926S, Cell Signaling), AKT (Catalog No.: 4691S, Cell Signaling), p-AKT (Catalog No.: 4060S, Cell Signaling), ERK (Catalog No.: SC-292838, Santa Cruz), p-ERK (Catalog No.: GTX129275, Genetex), JNK (Catalog No.: 474, Santa Cruz), p-JNK (Catalog No.: 9255S, Cell Signaling), MEK (Catalog No.: SC-56250, Santa Cruz), p-MEK (Catalog No.: 16500, Cell Signaling), P38 (Catalog No.: 110720, Genetex), p-P38 (Catalog No.: 133460, Genetex). After three washes for removing the residues of primary antibodies, and then incubated with HRP-conjugated goat anti-Rabbit IgG secondary antibody conjugated to horseradish peroxidase (Catalog No.: 31466, Thermo Fisher Scientific) at 1:5000 dilutions at RT for 1 h. The signals were detected on a charge-coupled device camera-based detection system (UVP Inc., Upland, CA, USA), and the ImageJ software (National Institutes of Health, USA) was used to quantify. The cropped images of blots shown in figures are used for illustrative purposes, full scan of the entire original gel(s) are included in the **Supplementary Figures**.

### Data analysis

All experiment results were expressed as the mean value and the standard deviation (S.D.) of three independent experiments with triplicate of each sample. Data were statistically analyzed by both Kruskal-Wallis and the ANOVA to determine whether statistically significant differences exist (P<0.05).

## Results

### Morphology and kinetic characterizations of CCDC-PA system

**Figure 1:**
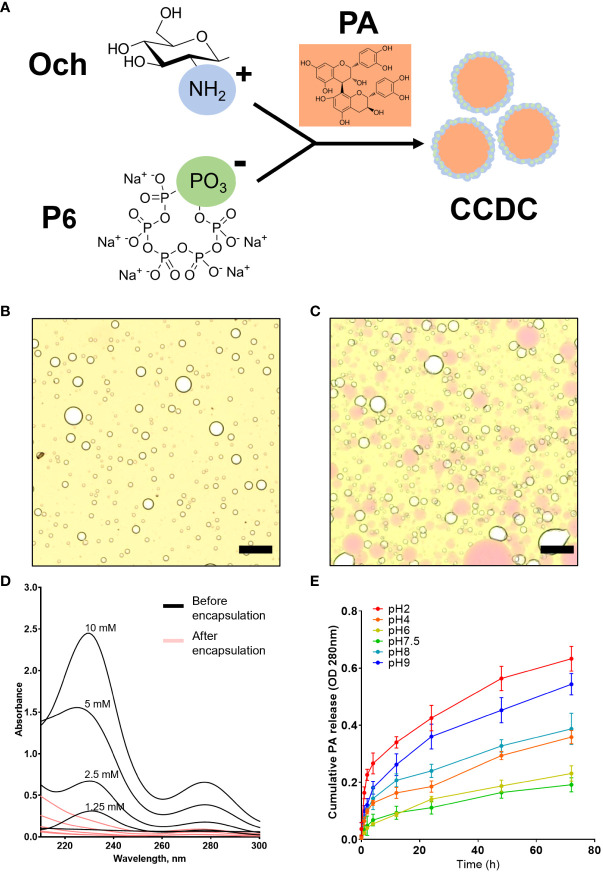
Structural and kinetic characterizations of CCDC-PA system. **(A)** The working model of CDCC formation by charge-charge interaction. **(B)** Multi-foamy structures of CCDC under a bright-field microscope at 10× magnification and **(C)** Encapsulation of PA by CCDC. Scale bars were 10 μm. **(D)** Encapsulation efficiency validated using UV-Vis spectrometry. **(E)** Profile of cumulative percentage of PA released from the CCDC system at various pH conditions for 24h.

Complex coacervates are a dense liquid phase formed by the associative separation of a mixture of oppositely charged polyelectrolytes (20). In this study, we found that positively charged chitosan (Och) interact with negatively charged P6 that formed complex coacervate phase to encapsulate proanthocyandies (PA) (**Figure 1A**). The bright-field microscope image indicated the formation of complex coacervates-based drug carrier, or simply CCDC, as characterized by the formation of spherical micron-sized droplets (**Figure 1B**). As shown in **Figure 1C**, grape seed PA, or simply PA (pink color), were successfully encapsulated by CCDC. To evaluate the encapsulation efficiency of PA in the CCDC system, the supernatant fraction (unencapsulated phase) of CDCC-PA was measured by UV spectrometer from 210 to 300 nm. Our results showed that a significant decreased of UV absorbance at 232 nm (specific to PA), indicating CDCC encapsulates PA with high yield (**Figure 1D**). To demonstrate the release profile of PA within the CCDC system, 1 mM PA was used as an example in this study. Our result showed that PA was steadily released from the CDCC system over time in a pH-dependent manner. Specifically, PA release was more stable at neutral pH 6.0 or 7.5 over 72 h period. In contrast, acidic (pH 2.0) or alkaline (pH 9.0) conditions led to a greater release of PA (**Figure 1E**). At higher pH conditions, Och tends to lose its positive charge due to deprotonation of amino groups which destabilize the ionic interaction within the CDCC system.

### CCDC-PA inhibited the cell proliferation of SCC4 cells

**Figure 2:**
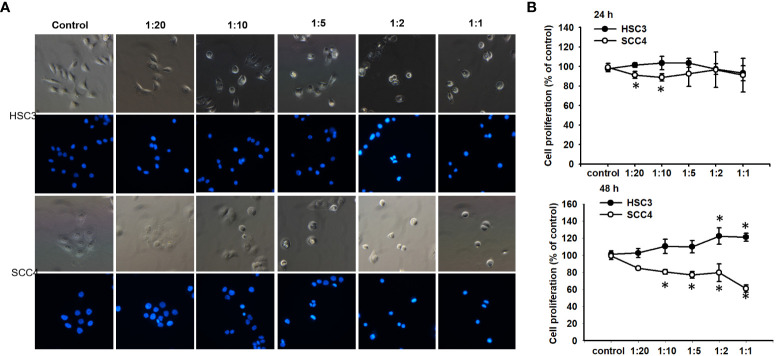
CCDC-PA inhibited the cell proliferation of SCC4 cells treated with CCDC-PA at various stoichiometric ratios at 24 h and 48 h. **(A)** Bright-field and immunofluorescence images of HSC-3 and SCC4 cells. **(B)** The cell proliferation of HSC-3 and SCC4 cells was determined by CCK-8 assay. Control indicates a condition without treatment of CCDC-PA. 1:20, 1:10, 1:5 1:2, and 1:1 indicate the final concentration of PA in the CCDC system is equivalent to 2.5, 5.0, 10, 25, and 50 µg/mL respectively. Data are expressed as mean ± S.D. (n = 3), with *P< 0.05 indicating the significant difference from the controls.

Although it has been demonstrated that PA inhibits the growth of cancer cells (10), the encapsulated form of PA and its effect on oral cancers remain uncharacterized. Therefore, the effect of CDCC-PA on the viability of both HSC-3 and SCC4 cells was evaluated using a CCK-8 assay. Both bright-field and immunofluorescence images visually showed that CCDC-PA can affect the cell proliferation of both HSC-3 and SCC4 cells in a dose-dependent manner at either 24 h or 48 h time point. In particular, the lower amount of CCDC-PA, the lesser inhibited proliferation of cancer cells (**Figure 2A**). However, the quantitative data using CCK-8 assay indicated that CCDC-PA only significantly inhibited the cell proliferation of SCC4 cells, yet HSC-3 cells. The dose-dependent trend of CCDC-PA in suppressing the cell proliferation of SCC4 cells was more evident at 48 h with PA concentrations of 5 µg/mL and higher (1:10 to 1:1) (**Figure 2B**). The CDCC system alone did not affect the proliferation of HSC-3 and SCC4 and therefore we confirmed that the cancer inhibition effect is from the PA released from the CDCC system.

### CCDC-PA inhibited the cell migration and invasion of HSC-3 and SCC4 cells

**Figure 3:**
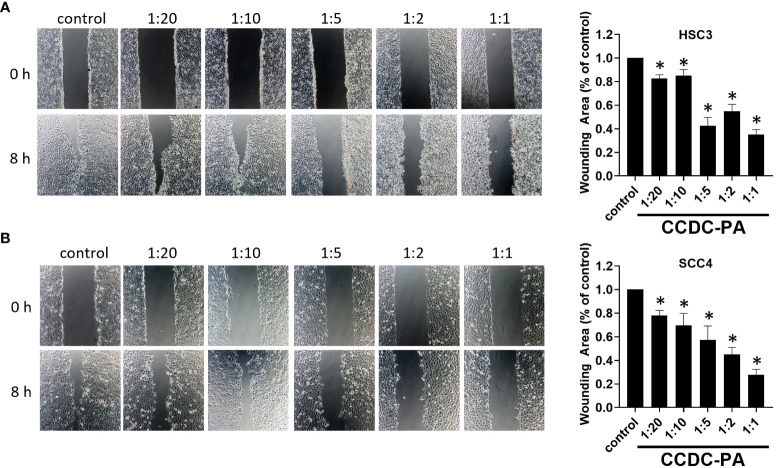
CCDC-PA inhibited the cell migration of HSC-3 and SCC4 cells treated with CCDC-PA at various stoichiometric ratios for 24 h. Wound healing assay of **(A)** HSC-3 and **(B)** SCC4 cells. Images of wound closure were taken at the baseline (0 h) and 8 h after wound generation. Representative photographs and quantitative data are graphically shown. Control indicates a condition without treatment of CCDC-PA. 1:20, 1:10, 1:5 1:2, and 1:1 indicate the final concentration of PA in the CCDC system is equivalent to 2.5, 5.0, 10, 25, and 50 µg/mL respectively. Data are expressed as mean ± S.D. (n = 3), with *P< 0.05 indicating the significant difference from the controls.

**Figure 4:**
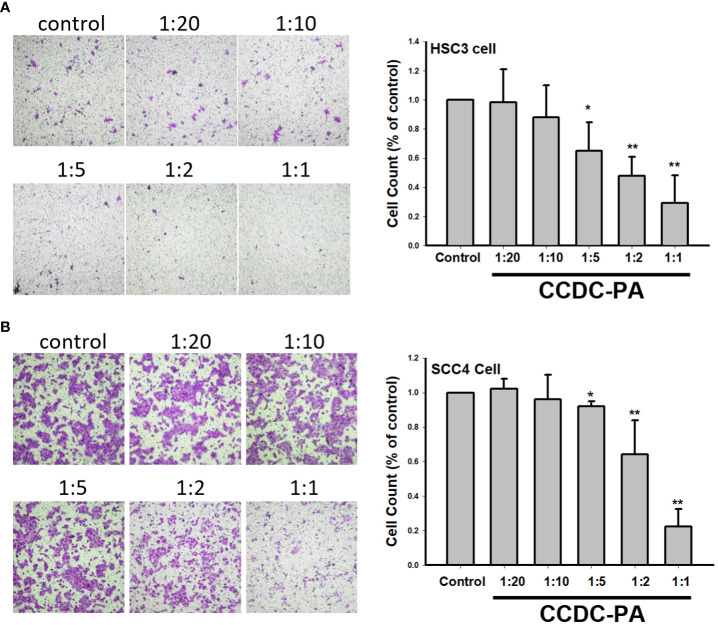
CCDC-PA inhibited the cell invasion of HSC-3 and SCC4 cells treated with CCDC-PA at various stoichiometric ratios for 24 h. Transwell Matrigel invasion assay of **(A)** HSC-3 and **(B)** SCC4 cells. Representative photographs and quantitative data are shown. Control indicates a condition without treatment of CCDC-PA. 1:20, 1:10, 1:5 1:2, and 1:1 indicate the final concentration of PA in the CCDC system is equivalent to 2.5, 5.0, 10, 25, and 50 µg/mL respectively. Data are expressed as mean ± S.D., with *P< 0.05 and **P< 0.01 indicating the significant difference from the controls.

As shown in **Figure 3A**, both representative photographs and quantitative data from the wound healing assay demonstrated that CCDC-PA can significantly impede the cell migration of HSC-3 cells in a dose-dependent manner. In particular, CCDC-PA at the stoichiometric ratio 1:1 (PA at 50 µg/mL) yielded the highest inhibitory effect. Similar results were also shown in SCC4 cells (**Figure 3B**). As shown in **Figure 4A**, both representative photographs and quantitative data from the Transwell Matrigel invasion assay suggested that CCDC-PA can markedly suppress the cell invasion of HSC-3 cells in a dose-dependent manner, whose highest inhibitory effect was at the stoichiometric ratio 1:1. Similar results were also observed in SCC4 cells (**Figure 4B**). We also confirmed that the inhibitory effect of CCDC-PA is mainly came from PA rather than the components (i.e. Och and P6) presented in the CCDC (**Supplemental Results**).

### CCDC-PA inhibited the expression of MMPs in HSC-3 and SCC4 cells

**Figure 5:**
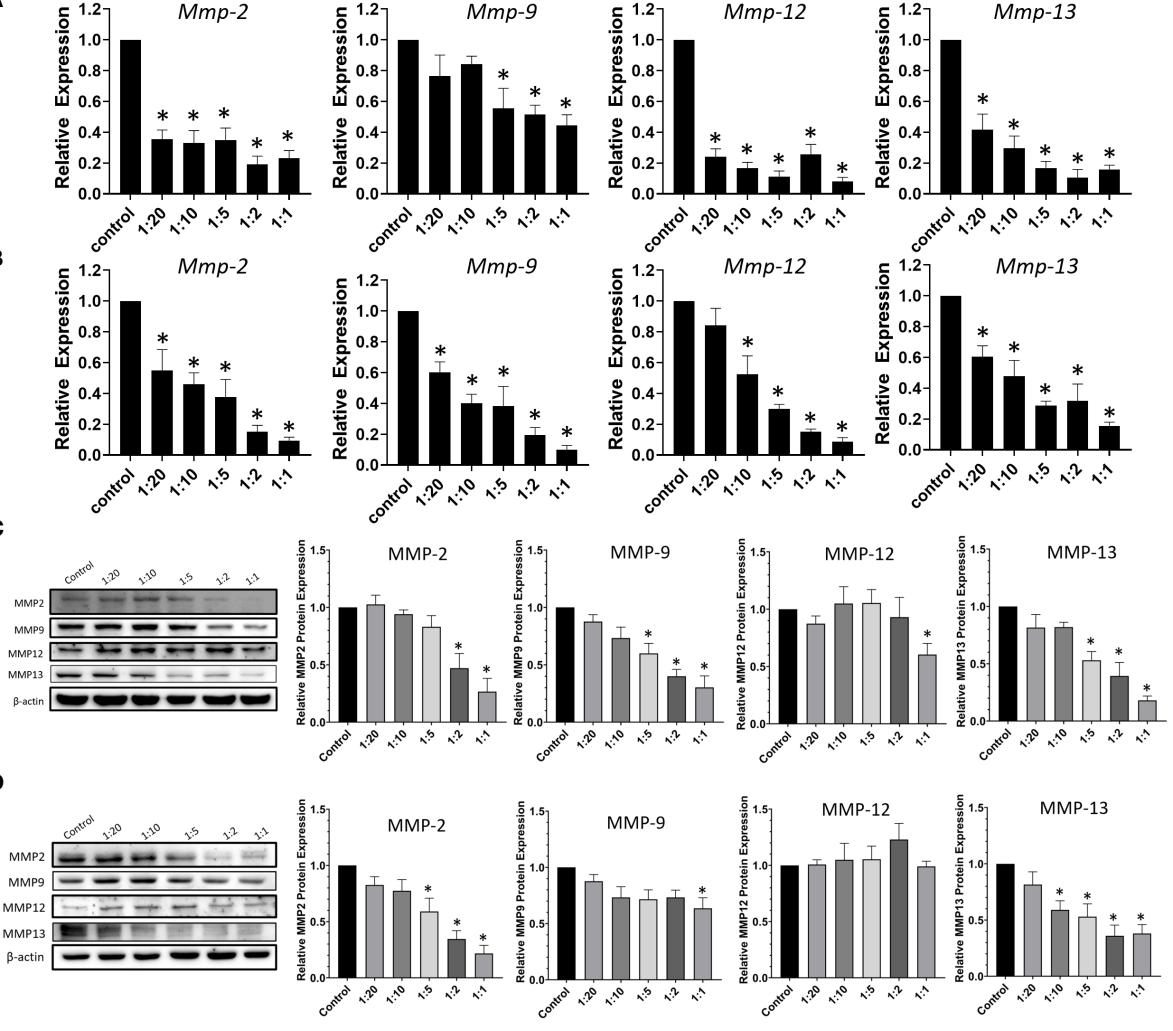
CCDC-PA inhibited the expression of MMPs in HSC-3 and SCC4 cells treated with CCDC-PA at various stoichiometric ratios for 24 h. The gene expression of *Mmp-2*, *Mmp-9*, *Mmp-12*, and *Mmp-13* was analyzed by real-time PCR in **(A)** HSC-3 and **(B)** SCC4 cells. The protein expression of MMP-2, MMP-9, MMP-12, and MMP-13 was observed by immunoblotting assay in **(C)** HSC-3 and **(D)** SCC4 cells. Control indicates a condition without treatment of CCDC-PA. 1:20, 1:10, 1:5 1:2, and 1:1 indicate the final concentration of PA in the CCDC system is equivalent to 2.5, 5.0, 10, 25, and 50 µg/mL respectively. The cropped images of blots shown in figures are used for illustrative purposes, full scan of the entire original gel(s) are included in the **Supplementary Figures**. Data are expressed as mean ± S.D. (n = 3), with *P< 0.05 indicating the significant difference from the controls.

Tumor invasion and metastasis require a profound remodeling and degradation of extracellular matrix (ECM), and MMPs present a family of ECM-degrading enzymes (21). MMPs can be classified into collagenases, gelatinases, and many more depending on their substrate specificity. Collagenases, such as MMP-13, are secreted enzymes capable of cleaving various types of collagen before degradation by other MMPs (22). This subfamily of MMPs is considered to associate with angiogenesis (23). Gelatinases, including MMP-2 and MMP-9, are another subfamily of MMPs that is well-established as important markers in the malignant progression of oral cancer (21). To examine the anti-metastatic potential of our experimental drug delivery system, the effects of CCDC-PA on the expression of different members of MMPs were assessed. As shown in **Figure 5A**, CCDC-PA was able to markedly reduce the gene expression of all Mmps tested (*Mmp-2*, *Mmp-9*, *Mmp-12* and *Mmp-13*) in HSC-3 cells in a dose-dependent manner. Similar to HSC-3 cells, the gene expression of *Mmp-2, Mmp-9, Mmp-12* and *Mmp-13* in SCC4 cells, in general, were strongly alleviated by CCDC-PA at every stoichiometric ratio (**Figure 5B**). The protein expression of these MMPs were further investigated by immunoblotting assay. As shown in **Figure 5C**, CCDC-PA was able to mitigate the expression of MMP-2, MMP-9, MMP-12, and MMP-13 in HSC-3 cells in a dose-dependent trend, in which the stoichiometric ratio 1:1 yielded the highest inhibitory effect. Similar results were also observed in SCC4 cells, except that MMP-12 expression was maintained as comparable as the control (**Figure 5D**).

### Inhibition of oral cancer cell migration by CCDC-PA is mediated *via* the PI3K/AKT signaling pathway

**Figure 6:**
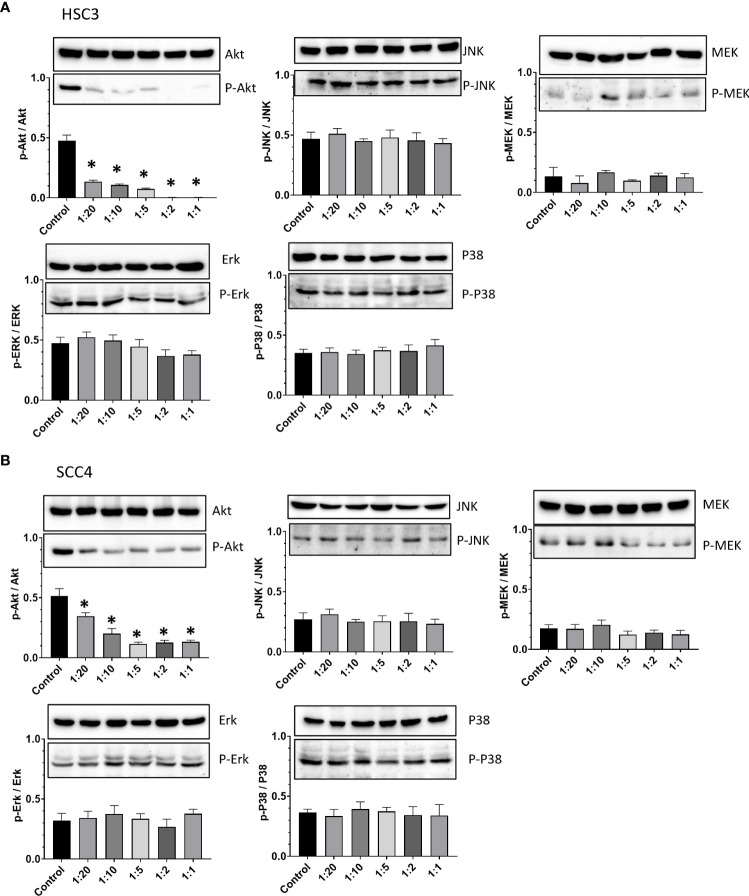
CCDC-PA inhibited the Akt phosphorylation in HSC-3 and SCC4 cells treated with CCDC-PA at various stoichiometric ratios for 24 h. Protein lysates were subjected to Western blot analysis, and β-actin was used as the loading control (data not shown). The protein expression of Akt, JNK, MEK, Erk, and p38 as well as their phosphorylated forms were observed in **(A)** HSC-3 and **(B)** SCC4 cells. The quantification of p-Akt/Akt, p-JNK/JNK, p-MEK/MEK, p-Erk/Erk, and p-p38/p38 ratios was further done by densitometry analysis in **(A)** HSC-3 and **(B)** SCC4 cells. Control indicates a condition without treatment of CCDC-PA. 1:20, 1:10, 1:5 1:2, and 1:1 indicate the final concentration of PA in the CCDC system is equivalent to 2.5, 5.0, 10, 25, and 50 µg/mL respectively. The cropped images of blots shown in figures are used for illustrative purposes, full scan of the entire original gel(s) are included in the **Supplementary Figures**. Data are expressed as mean ± S.D. (n = 3), with *P< 0.05 indicating the significant difference from the controls.

PI3K/Akt axis is one of crucial signaling pathways, which is considered as a master regulator of oral cancer progression. The activation of this pathway may contribute to cancer cell survival and growth, angiogenesis, and migration and invasion (24, 25). Another key signaling pathway in the oral carcinogenesis is MAPKs which comprise of three major signaling pathways termed c-Jun N-terminal kinase (JNK), extracellular signal-regulated kinase (Erk), and p38. The Erk signaling pathway consists of three components, among which is MEK (26). Considering the pivotal roles of these pathways during the course of oral cancer, the expression and phosphorylation of Akt, JNK, MEK, Erk, and p38 were studied to explore the possible mechanisms underlying the anticancer effects of CCDC-PA. As shown in **Figure 6**, CCDC-PA could significantly inhibit the phosphorylation of Akt, but not that of MAPKs, in both HSC-3 and SCC4 cells in a dose-dependent manner.

**Figure 7:**
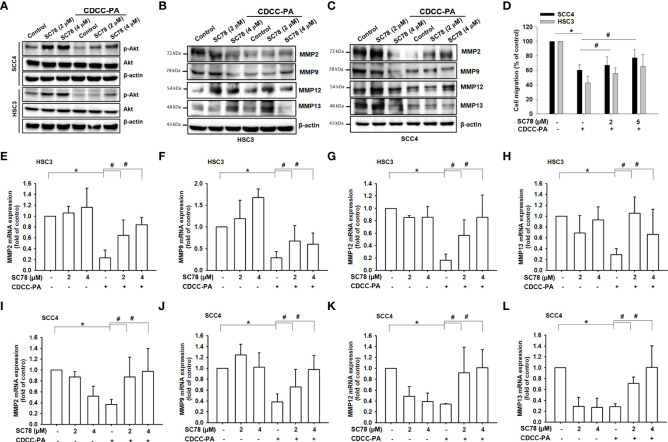
Inhibition of oral cancer cell migration by CCDC-PA is mediated *via* the PI3K/AKT signaling pathway. After cells treated with the AKT agonist SC78, the gene expression levels of *Mmp-2, Mmp-9, Mmp-12* and *Mmp-13* were analyzed in **(A–D)** HSC-3 cells and **(E–H)** SCC4 cells in response to CCDC-PA (1:5 ratio) at 24h. The protein expression levels of MMP-2, MMP-9, MMP-12 and MMP-13 were analyzed in **(I)** HSC-3 and **(J)** SCC4 cells in response to CCDC-PA at 24h. **(K)** The protein levels of AKT and phosphorylated forms were analyzed. **(L)** Cell migration assay of HSC-3 and SCC4 cells were analyzed, and quantified. Control indicates a condition without treatment of CCDC-PA. 1:20, 1:10, 1:5 1:2, and 1:1 indicate the final concentration of PA in the CCDC system is equivalent to 2.5, 5.0, 10, 25, and 50 µg/mL respectively. The cropped images of blots shown in figures are used for illustrative purposes, full scan of the entire original gel(s) are included in the **Supplementary Figures**. Data are presented as the mean ± standard deviation. * and # indicate P<0.05.

We then treated cells with SC78, an AKT agonist, to detect whether CCDC-PA at 1:5 ratio (a concentration that showed a significant effect in most results) inhibited oral cancer through the AKT signaling pathway. Our results demonstrated that treatment of SC78 promoted phosphorylation of AKT and reversed the CCDC-PA-induced suppression of AKT phosphorylation (**Figure 7A**) and MMPs (**Figures 7B, C, E–L**). Moreover, addition of SC78 enhanced cell migration in the presence of CCDC-PA (**Figure 7D**). These findings suggested that activation of Akt signaling pathway can be halted by our experimental delivery drug system to rescue the oral cancer progression.

## Discussion

Delivery and release of bioactive compounds by encapsulation is of great interest in the field of biomedicine (27). The inspiration behind the use of complex coacervates as a drug carrier in this study came from the underwater adhesive of sandcastle worms. Sandcastle worms are able to secrete and cure their adhesive under the sea through the mixture of oppositely charged proteins into a liquid complex coacervate phase (15). Having the same concept, in our study, CCDC was successfully formed through the complex coacervation of oppositely charged macromolecules derived from natural polysaccharides. Considering the natural origin of its components, CCDC was presumed to be compatible to biological systems. In consistency with the literature (17, 28), we observed that the formation of complex coacervation significantly depended on the conditions including pH and molar ratio of charged macromolecules. The optimal condition for complex coacervation was thus established at pH 7.5 with 1:1 ratio of Och and P6 in our experiments.

The benefits of dietary intake of polyphenolic compounds in preventing and treating diseases have been recognized for their wide range of biological activities (5, 6). Flavonoids comprise the main group of polyphenols abundantly found in the nature (6), and grape seed PA are among the most common (7). Although flavonoids possess promising health benefits, their weak stability and low insolubility are obstacles to their bioaccessibility. Moreover, flavonoids are degraded in the extreme acidic pH of gastric juice, compromising their bioavailability (27). Therefore, encapsulation can be an effective approach to protect these compounds with lower doses to achieve therapeutic effects. Although complex coacervation is one of physicochemical methods for encapsulation techniques (29), encapsulation of proteins within a coacervate phase can represent a challenge because not all proteins of interest are strongly charged (30). Intriguingly, our study the first time provided a key step towards the preparation of CDCC formulated from complex coacervates to successfully encapsulate grape seed PA for the purpose of oral cancer treatment. Importantly, the release of PA from drug carriers can be tuned by the changes of pH. This mode of action for cargo releasing can be advantageous for clinical application since it can trigger the disassembly of complex coacervates-based delivery vehicles in human bodily fluids (31).

Oral cancer, dominated up to 90% by OSCC, is a significant health burden in the world (1, 2). It is crucial to explore novel and effective strategies for the prevention and treatment of this lethal disease. Previous studies indicated the chemopreventive effects of grape seed PA on different OSCC models both *in vitro* and *in vivo* (10, 11). At the cellular level, anticancer effects of PA include the inhibition of cell survival and proliferation, induction of apoptosis and cell cycle arrest, and suppression of metastatic processes of cell migration and invasion (8–11). In addition, the selective cytotoxicity of grape seed PA to human OSCC cells was also reported (32). These findings collectively suggest that grape seed PA can efficiently impair OSCC cells without damaging normal cells. However, their efficacy in clinical practice can be limited due to their insufficient biodistribution and bioavailability (7). To overcome these weaknesses, in our study, CCDC was synthesized to encapsulate grape seed PA (CCDC-PA) for preserving their integrity and bioactivity until they reach the cancer cells. Our data demonstrated that CCDC-PA was able to inhibit the cell proliferation of SCC4 cells in a dose-dependent manner. In particular, the smaller stoichiometric ratio between CCDC and PA, or the lower molar concentration of PA, the lesser proliferation of cancer cells. These findings implicated the improvement of PA effectiveness in treating oral cancer following the encapsulation and delivery by CCDC. As opposed to SCC4 cells, HSC-3 cells were not significantly responsive to CCDC-PA. This low responsiveness to treatment may be due to the metastatic nature of HSC-3 cells. However, we found that CCDC-PA efficiently inhibited the cell migration and invasion in both cell lines. The same dose-dependent manner was also observed, suggesting the benefits of CCDC to enhance the chemopreventive effects of PA for inhibiting OSCC progression.

The metastatic potential of OSCC mainly relies on its ability to degrade the ECM for penetrating the basement membrane, invading the adjacent tissues, and initiating tumor angiogenesis. Degradation of ECM generally requires the activities of MMPs (21–23). The roles of MMPs in tumor progression, tumor angiogenesis, and metastatic dissemination have been reported in the literature. It was indicated that MMP-2, MMP-9, and MMP-13 are among the crucial regulators in the progression and invasion of OSCC, and they can serve as reliable markers of these phenomena (33). An *in vitro* study showed the inhibitory effects of grape seed PA on the secretion of MMP-2 and MMP-9 in the tongue squamous cell carcinoma cell line Tca8113, indicating the anti-metastatic capability of PA (10). In this study, our results also observed that CCDC-PA can reduce both gene levels and protein expression of MMP-2, MMP-9, and MMP-13 in HSC-3 and SCC4 cells in a dose-dependent trend, in which the stoichiometric ratio 1:20 (PA concentration at 2.5 µg/mL) already showed inhibitory effects as compared to a previous study used unprotected PA at 25 µg/mL (10). Our results implied that protected PA using the CCDC system may provide a more potent anticancer effect using lower doses. As many MMPs, macrophage elastase called MMP-12 is able to degrade ECM components to contribute to remodeling processes as well (34). Since MMP-12 is mainly expressed on macrophages, it is not surprising that we did not observe any impact of CCDC-PA on MMP-12 protein expression in SCC4 cells. However, MMP-12 expression in HSC-3 cells was somewhat affected by CCDC-PA, possibly due to its origin of metastatic lymph nodes. Taken together, our results proposed that the anti-metastatic potential of grape seed PA in OSCC can be improved after encapsulation and delivery by CCDC.

Chemopreventive agents that interfere with a particular pathway or a complex network specifically involved in the tumorigenesis may be more beneficial for cancer treatment (35). The PI3K/Akt pathway is well-known as a major signaling pathway that controls many cellular processes such as proliferation, apoptosis, angiogenesis, and metastasis during the progression of many cancers (24). Being a commonly upregulated pathway in oral cancer, targeting PI3K/Akt axis might be helpful in the prevention and treatment of this disease (25). Previous data indicated that grape seed PA can suppress the phosphorylation of Akt which is central to PI3K/Akt pathway, compromising downstream cascades to prevent the progression of tongue squamous cell carcinoma cells (10). Similarly, we also found that Akt phosphorylation was significantly inhibited by CCDC-PA in HSC-3 and SCC4 cells in a dose-dependent manner, indicating the importance of Akt signaling in the mode of action of CCDC-PA for inhibiting OSCC development. In addition to PI3K/Akt, MAPKs pathway consisting of Erk, JNK, and p38 subfamily also presents another important mechanism for oral carcinogenesis (26). A few evidences have reported that grape seed PA can exert contradictory effects on the activation of these MAPKs (36–38). However, in our study, none of three pathways was affected by CCDC-PA. This may be ascribed to the differences in the cancer types and experimental models among studies as well as the dose-dependent effects of PA itself.

In summary, our *in vitro* results support the concept that grape seed PA encapsulated by complex coacervates-based drug delivery can act as an effective anticancer agent for patients with oral cancer. However, some limitations still remain. Due to the static nature of *in vitro* experiments, our data cannot fully represent the bioaccessibility and bioavailability of CCDC-PA in *in vivo* tissues and human body. Therefore, more animal studies and clinical trials are required to explore the distribution, digestion, and absorption of this drug delivery system for their biomedical applications in the future.

## Data availability statement

The original contributions presented in the study are included in the article/**Supplementary Material**. Further inquiries can be directed to the corresponding author.

## Author contributions

C-SW and YW contributed to the conception and wrote the article. C-SW and YW performed the characterization of the CDCC system. J-FL, YW, S-DL, S-FC performed the *in vitro* experiments. J-FL, H-MH, S-DL, J-HL, and S-WF analyzed the data and provided advice. All authors contributed to the article and approved the submitted version.

## Funding

This research was funded by the Ministry of Science and Technology (MOST), Taiwan, grant number 110-2320-B-038-015 and TMU Research Center of Cancer Translational Medicine from The Featured Areas Research Center Program within the framework of the Higher Education Sprout Project by the Ministry of Education (MOE) in Taiwan, Grant/Award Numbers: DP2-111-21121-01-O-10-03.

## Conflict of interest

The authors declare that the research was conducted in the absence of any commercial or financial relationships that could be construed as a potential conflict of interest.

## Publisher’s note

All claims expressed in this article are solely those of the authors and do not necessarily represent those of their affiliated organizations, or those of the publisher, the editors and the reviewers. Any product that may be evaluated in this article, or claim that may be made by its manufacturer, is not guaranteed or endorsed by the publisher.

## References

[1] Ren Z-H Hu C-Y He H-R Li Y-J Lyu J. Global and regional burdens of oral cancer from 1990 to 2017: Results from the global burden of disease study. Cancer Commun (Lond) (2020) 40:81–92. doi: 10.1002/cac2.12009 32067418 PMC7163731

[2] Sung H Ferlay J Siegel RL Laversanne M Soerjomataram I Jemal A. Global cancer statistics 2020: GLOBOCAN estimates of incidence and mortality worldwide for 36 cancers in 185 countries. CA: A Cancer J Clin (2021) 71:209–49. doi: 10.3322/caac.21660 33538338

[3] Chinn SB Myers JN. Oral cavity carcinoma: Current management, controversies, and future directions. J Clin Oncol (2015) 33:3269–76. doi: 10.1200/JCO.2015.61.2929 PMC5320919 26351335

[4] Saka-Herrán C Jané-Salas E Mari-Roig A Estrugo-Devesa A López-López J. Time-to-Treatment in oral cancer: Causes and implications for survival. Cancers (Basel) (2021) 13:1321. doi: 10.3390/cancers13061321 33809427 PMC8000007

[5] Bernardini S Tiezzi A Laghezza Masci V Ovidi E. Natural products for human health: an historical overview of the drug discovery approaches. Natural Product Res (2017) 32:1926–50. doi: 10.1080/14786419.2017.1356838 28748726

[6] Panche AN Diwan AD Chandra SR. Flavonoids: an overview. J Nutr Sci (2016) 5:e47–7. doi: 10.1017/jns.2016.41 PMC5465813 28620474

[7] Unusan N. Proanthocyanidins in grape seeds: An updated review of their health benefits and potential uses in the food industry. J Funct Foods (2020) 67:103861. doi: 10.1016/j.jff.2020.103861

[8] Antonacci D Cucina A Antonacci D Bizzarri M. Anticancer effects of grape seed extract on human cancers: A review. J Carcinogenesis Mutagenesis (2014) 8:1–14. doi: 10.4172/2157-2518.s8-005

[9] Ravindranathan P Pasham D Balaji U Cardenas J Gu J Toden S. Mechanistic insights into anticancer properties of oligomeric proanthocyanidins from grape seeds in colorectal cancer. Carcinogenesis (2018) 39:767–77. doi: 10.1093/carcin/bgy034 PMC5972632 29684110

[10] Yang N Gao J Cheng X Hou C Yang Y Qiu Y. Grape seed proanthocyanidins inhibit the proliferation, migration and invasion of tongue squamous cell carcinoma cells through suppressing the protein kinase b/nuclear factor-κB signaling pathway. Int J Mol Med (2017) 40:1881–8. doi: 10.3892/ijmm.2017.3162 PMC5716438 29039443

[11] Lee Y. Cancer chemopreventive potential of procyanidin. Toxicol Res (2017) 33:273–82. doi: 10.5487/TR.2017.33.4.273 PMC5654195 29071011

[12] Coelho JF Ferreira PC Alves P Cordeiro R Fonseca AC Góis JR. Drug delivery systems: Advanced technologies potentially applicable in personalized treatments. EPMA J (2010) 1:164–209. doi: 10.1007/s13167-010-0001-x 23199049 PMC3405312

[13] Wang CS Stewart RJ. Localization of the bioadhesive precursors of the sandcastle worm, phragmatopoma californica (Fewkes. J Exp Biol (2012) 215:351–61. doi: 10.1242/jeb.065011 22189779

[14] Wang CS Stewart RJ. Multipart copolyelectrolyte adhesive of the sandcastle worm, phragmatopoma californica (Fewkes): catechol oxidase catalyzed curing through peptidyl-DOPA. Biomacromolecules (2013) 14:1607–17. doi: 10.1021/bm400251k 23530959

[15] Stewart RJ Wang CS Song IT Jones JP. The role of coacervation and phase transitions in the sandcastle worm adhesive system. Adv colloid Interface Sci (2017) 239:88–96. doi: 10.1016/j.cis.2016.06.008 27393642 PMC5182194

[16] Johnson NR Wang Y. Coacervate delivery systems for proteins and small molecule drugs. Expert Opin Drug Deliv (2014) 11:1829–32. doi: 10.1517/17425247.2014.941355 PMC4617778 25138695

[17] Tiwari P Bharti I Bohidar HB Quadir S Joshi MC Arfin N. Complex coacervation and overcharging during interaction between hydrophobic zein and hydrophilic laponite in aqueous ethanol solution. ACS Omega (2020) 5:33064–74. doi: 10.1021/acsomega.0c04647 PMC7774070 33403268

[18] Senapati S Mahanta AK Kumar S Maiti P. Controlled drug delivery vehicles for cancer treatment and their performance. Signal Transduct Target Ther (2018) 3:7–7. doi: 10.1038/s41392-017-0004-3 29560283 PMC5854578

[19] Elaiwy O El Ansari W AlKhalil M Ammar A. Epidemiology and pathology of oral squamous cell carcinoma in a multi-ethnic population: Retrospective study of 154 cases over 7 years in Qatar. Ann Med Surg (Lond) (2020) 60:195–200. doi: 10.1016/j.amsu.2020.10.029 33163176 PMC7610004

[20] Kim HJ Yang B Park TY Lim S Cha HJ. Complex coacervates based on recombinant mussel adhesive proteins: their characterization and applications. Soft Matter (2017) 13:7704–16. doi: 10.1039/c7sm01735a 29034934

[21] Singh RD Haridas N Patel JB Shah FD Shukla SN Shah PM. Matrix metalloproteinases and their inhibitors: correlation with invasion and metastasis in oral cancer. Indian J Clin Biochem (2010) 25:250–9. doi: 10.1007/s12291-010-0060-8 PMC3001841 21731196

[22] Gkouveris I Nikitakis N Aseervatham J Rao N Ogbureke K. Matrix metalloproteinases in head and neck cancer: current perspectives. Metalloproteinases In Med (2017) 4:47–61. doi: 10.2147/mnm.s105770

[23] Quintero-Fabián S Arreola R Becerril-Villanueva E Torres-Romero JC Arana-Argáez V Lara-Riegos J. Role of matrix metalloproteinases in angiogenesis and cancer. Front Oncol (2019) 9:1370. doi: 10.3389/fonc.2019.01370 31921634 PMC6915110

[24] Yang J Nie J Ma X Wei Y Peng Y Wei X. Targeting PI3K in cancer: mechanisms and advances in clinical trials. Mol Cancer (2019) 18:26–6. doi: 10.1186/s12943-019-0954-x PMC6379961 30782187

[25] Harsha C Banik K Ang HL Girisa S Vikkurthi R Parama D. Targeting AKT/mTOR in oral cancer: Mechanisms and advances in clinical trials. Int J Mol Sci (2020) 21:3285. doi: 10.3390/ijms21093285 PMC7246494 32384682

[26] Peng Q Deng Z Pan H Gu L Liu O Tang Z. Mitogen-activated protein kinase signaling pathway in oral cancer. Oncol Lett (2018) 15:1379–88. doi: 10.3892/ol.2017.7491 PMC5776165 29434828

[27] Yousefi M Shadnoush M Sohrabvandi S Khorshidian N Mortazavian AM. Encapsulation systems for delivery of flavonoids: A review. Biointerface Res Appl Chem (2021) 11:13934–51. doi: 10.33263/briac116.1393413951

[28] Kaushik P Rawat K Aswal VK Kohlbrecher J Bohidar HB. Mixing ratio dependent complex coacervation versus bicontinuous gelation of pectin with *in situ* formed zein nanoparticles. Soft Matter (2018) 14:6463–75. doi: 10.1039/c8sm00809d 30051132

[29] Trojanowska A Nogalska A Valls RG Giamberini M Tylkowski B. Technological solutions for encapsulation. Polymer Eng (2017) 2(9):171–202. doi: 10.1515/9783110469745-006

[30] Kapelner RA Obermeyer AC. Ionic polypeptide tags for protein phase separation. Chem Sci (2019) 10:2700–7. doi: 10.1039/c8sc04253e PMC6419950 30996987

[31] Blocher WC Perry SL. Complex coacervate-based materials for biomedicine. WIREs Nanomedicine Nanobiotechnology (2016) 8:1–28. doi: 10.1002/wnan.1442 27813275

[32] Schuck AG. Selective cytotoxicity of a grape seed proanthocyanidin extract to human oral carcinoma HSC-2 cells. Cell Dev Biol (2013) 2(3):1–8. doi: 10.4172/2168-9296.1000121

[33] Mishev G Deliverska E Hlushchuk R Velinov N Aebersold D Weinstein F. Prognostic value of matrix metalloproteinases in oral squamous cell carcinoma. Biotechnol Biotechnol Equip (2014) 28:1138–49. doi: 10.1080/13102818.2014.967510 PMC4433935 26019601

[34] Lagente V Le Quement C Boichot E. Macrophage metalloelastase (MMP-12) as a target for inflammatory respiratory diseases. Expert Opin Ther Targets (2009) 13:287–95. doi: 10.1517/14728220902751632 19236151

[35] Sarkar FH Li Y. Targeting multiple signal pathways by chemopreventive agents for cancer prevention and therapy. Acta Pharmacologica Sin (2007) 28:1305–15. doi: 10.1111/j.1745-7254.2007.00689.x 17723164

[36] Wang L Zhan J Huang W. Grape seed proanthocyanidins induce apoptosis and cell cycle arrest of HepG2 cells accompanied by induction of the MAPK pathway and NAG-1. Antioxidants (Basel) (2020) 9:1200. doi: 10.3390/antiox9121200 PMC7760884 33260632

[37] Zheng W Feng Y Bai Y Feng Z Yang X Dang B. Proanthocyanidins extracted from grape seeds inhibit the growth of hepatocellular carcinoma cells and induce apoptosis through the MAPK/AKT pathway. Food Bioscience (2022) 45:101337. doi: 10.1016/j.fbio.2021.101337

[38] Xu Y Huang Y Chen Y Cao K Liu Z Wan Z. Grape seed proanthocyanidins play the roles of radioprotection on normal lung and radiosensitization on lung cancer *via* differential regulation of the MAPK signaling pathway. J Cancer (2021) 12:2844–54. doi: 10.7150/jca.49987 PMC8040900 33854585

