# Supplementary material for: Proanthocyanidins-loaded complex coacervates-based drug delivery attenuates oral squamous cell carcinoma cells metastatic potential through down-regulating the Akt signaling pathway
Source: Front Oncol. 2022 Oct 18;12:1001126. doi: 10.3389/fonc.2022.1001126 (PMC9623311; doi:10.3389/fonc.2022.1001126)
Supplement: Supplementary file 1 [file DataSheet_1.pdf]

Supplemental Results

Title: Proanthocyanidins-loaded complex coacervates-based drug delivery attenuates oral squamous cell carcinoma cells metastatic potential through down-regulating the Akt signaling pathway

Authors: Ju-Fang Liu1, Yinshen Wee2, Shen-Dean Luo3, Shwu-Fen Chang4, Shihai Jia5, Sheng-Wei Feng6, Huei-Mei Huang4, Jiann-Her Lin7 and Ching-Shuen Wang6*

1 School of Oral Hygiene, College of Oral Medicine, Taipei Medical University, Taipei 11031, Taiwan.

2 Department of Pathology, University of Utah, Salt Lake City, UT 84112, USA.

3 Department of Otolaryngology, Kaohsiung Chang Gung Memorial Hospital and Chang Gung University College of Medicine, Kaohsiung 833, Taiwan.

4 Graduate Institute of Medical Sciences, College of Medicine, Taipei Medical University, Taipei, Taiwan.

5 Department of Neurobiology, University of Utah, Salt Lake City, UT 84112, USA.

6 School of Dentistry, College of Oral Medicine, Taipei Medical University, Taipei, Taiwan.

7 Department of Neurosurgery, Taipei Medical University Hospital, Taipei 11031, Taiwan.

*** Correspondence:**
Corresponding Author Correspondence: chingshuenwang@tmu.edu.tw; Tel.: +886-0227361661

Western blot images of HSC3 cells related experiments

These original western blot images are related to Fig 5C in the main text.

HSC3-MMP2 (N=5)

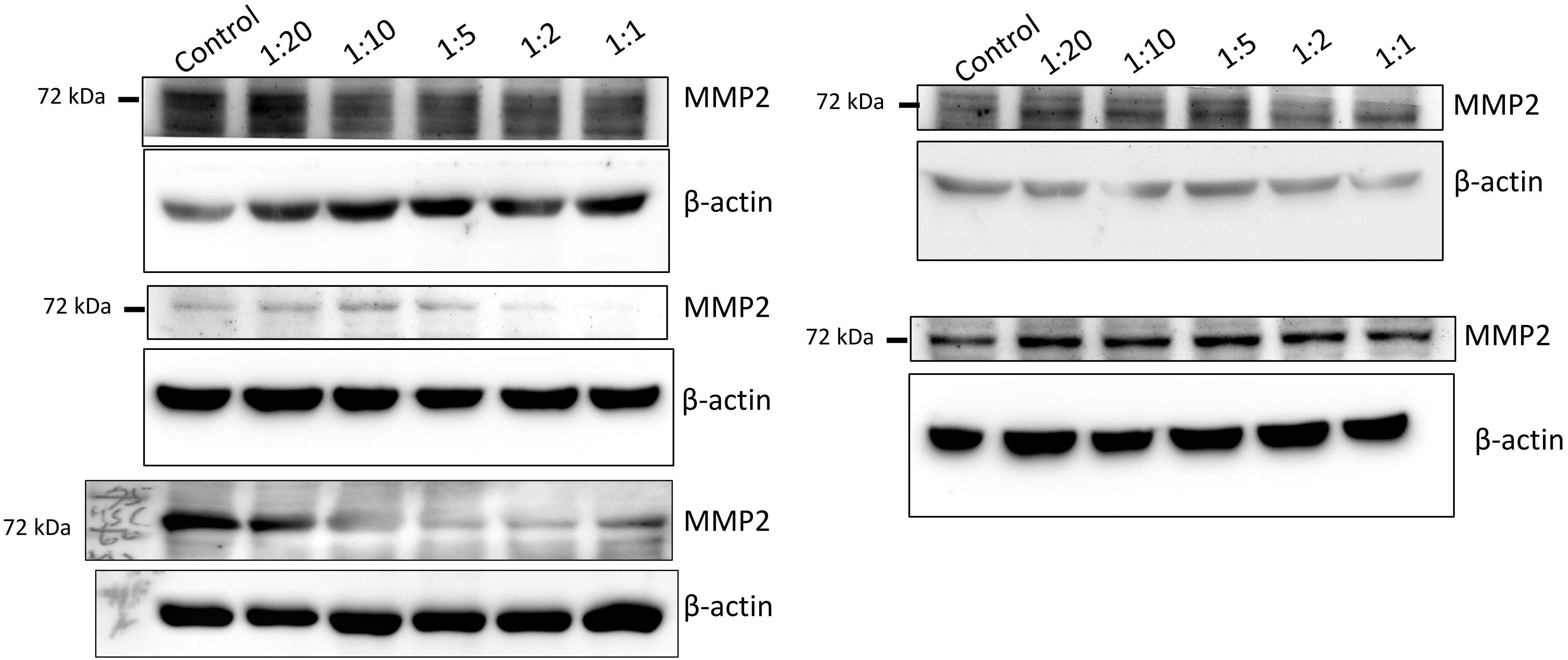

These original western blot images are related to Fig 5C in the main text.

HSC-MMP9 (N=6)

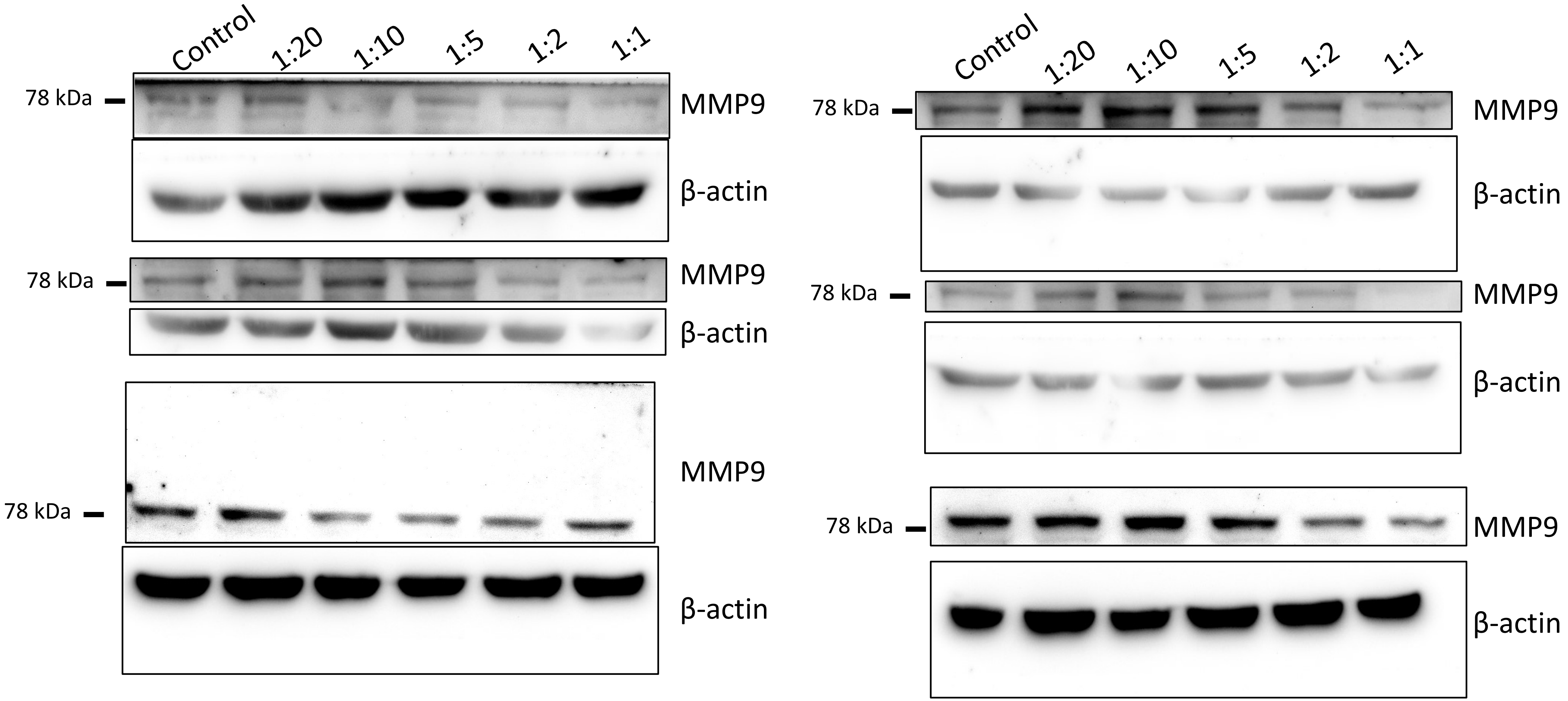

These original western blot images are related to Fig 5C in the main text.

HSC-MMP12 (N=5)

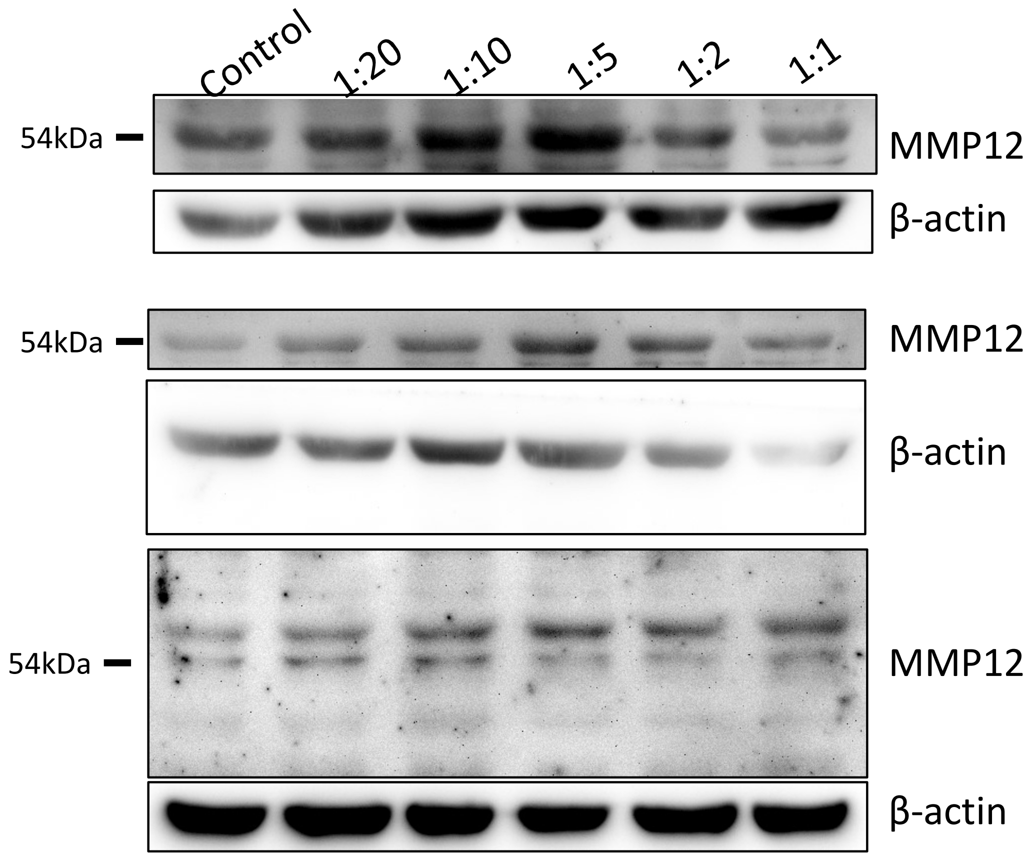

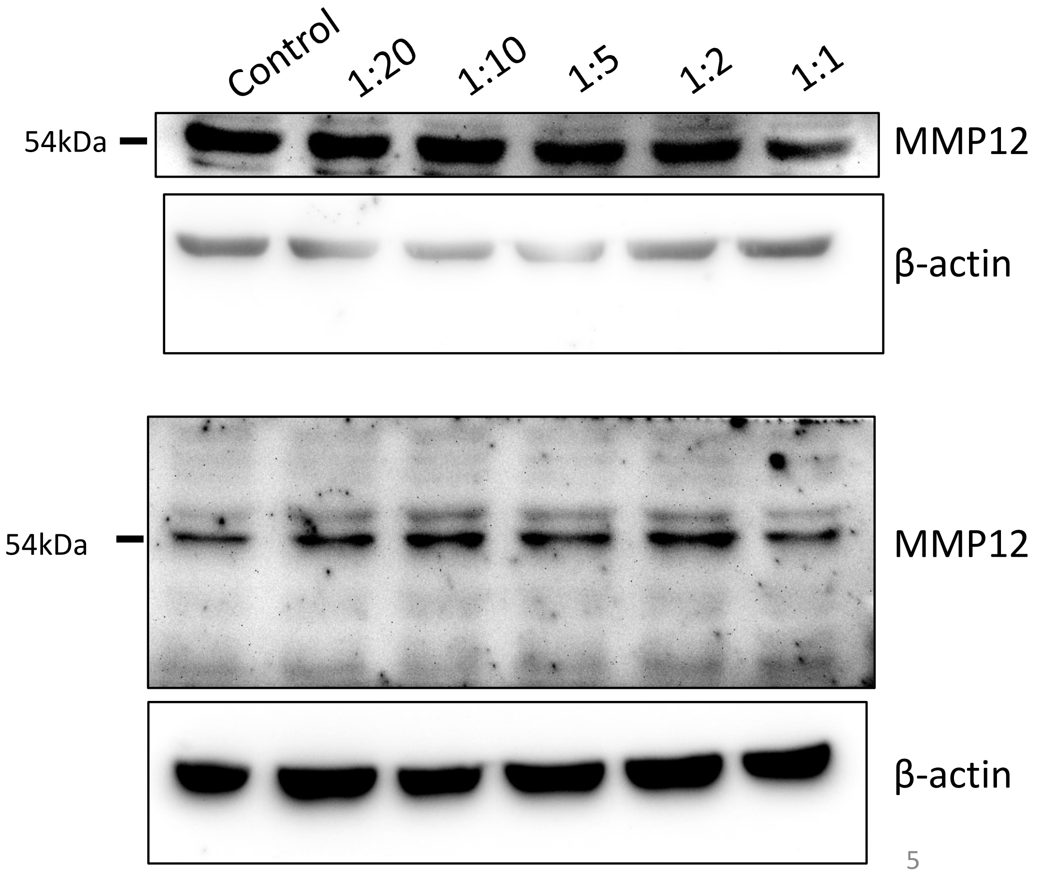

These original western blot images are related to Fig 5C in the main text.

HSC-MMP13 (N=5)

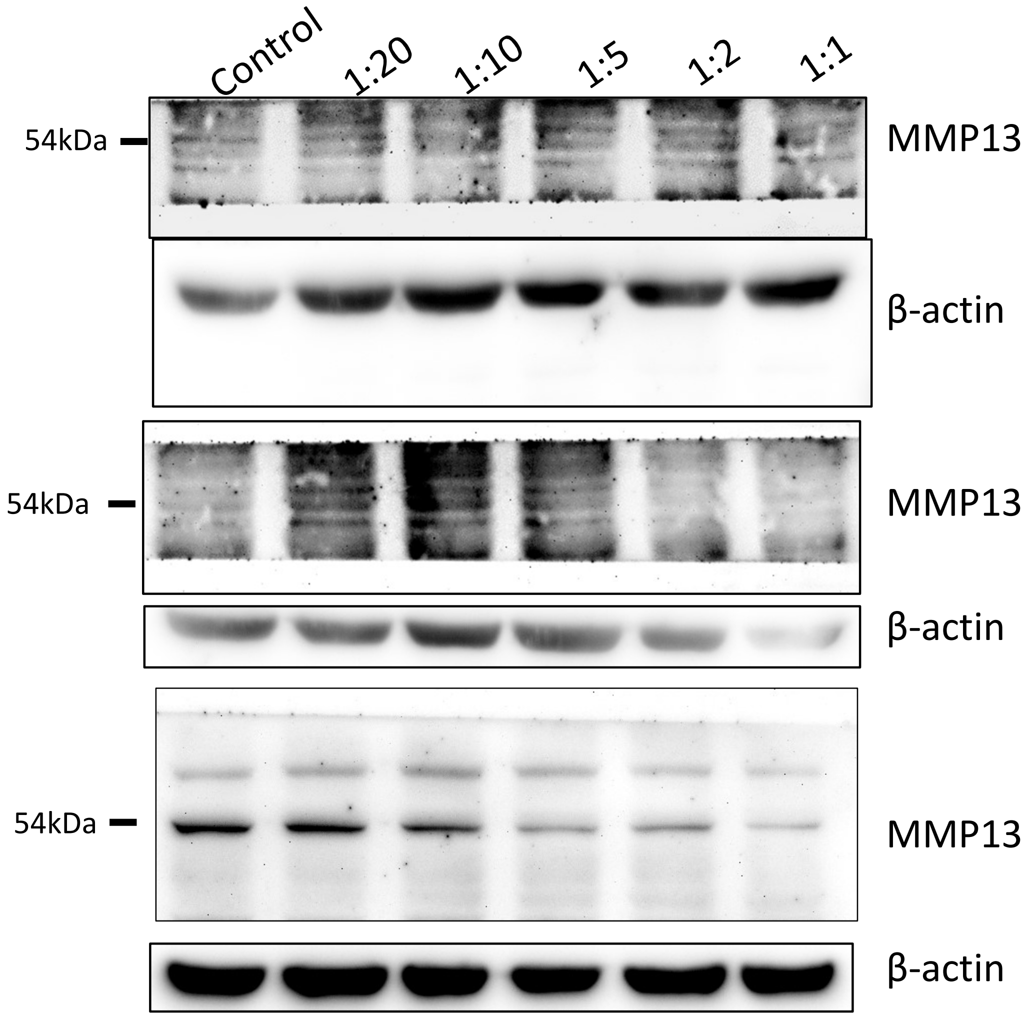

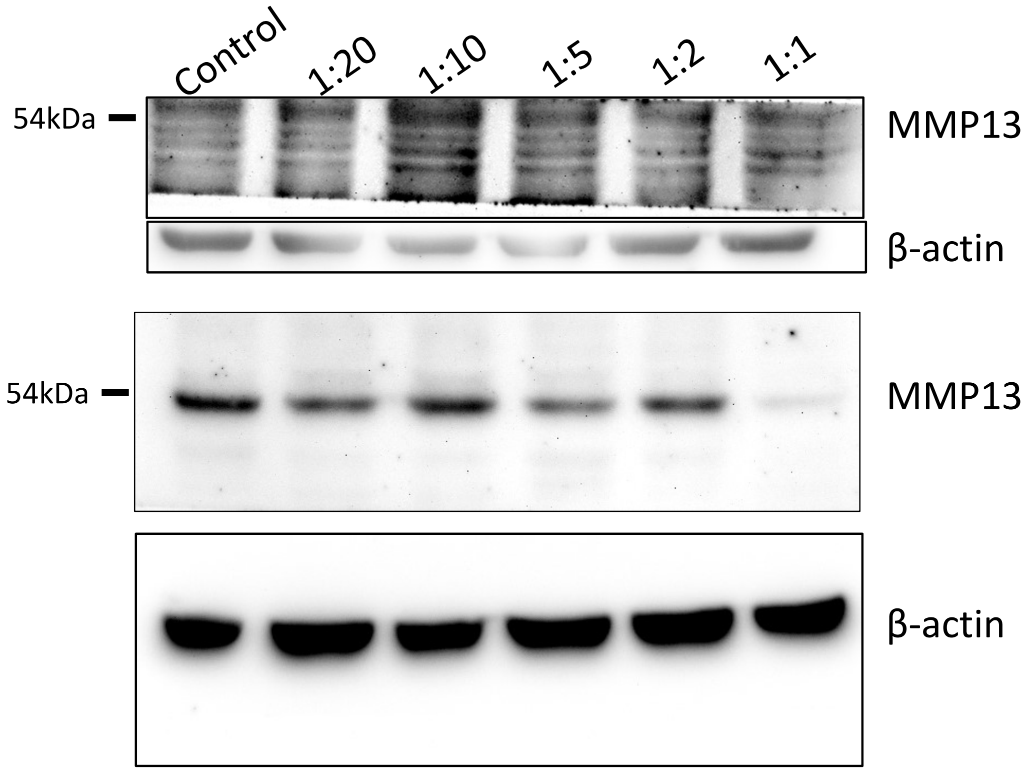

HSC3 p-JNK, JNK (N=8)

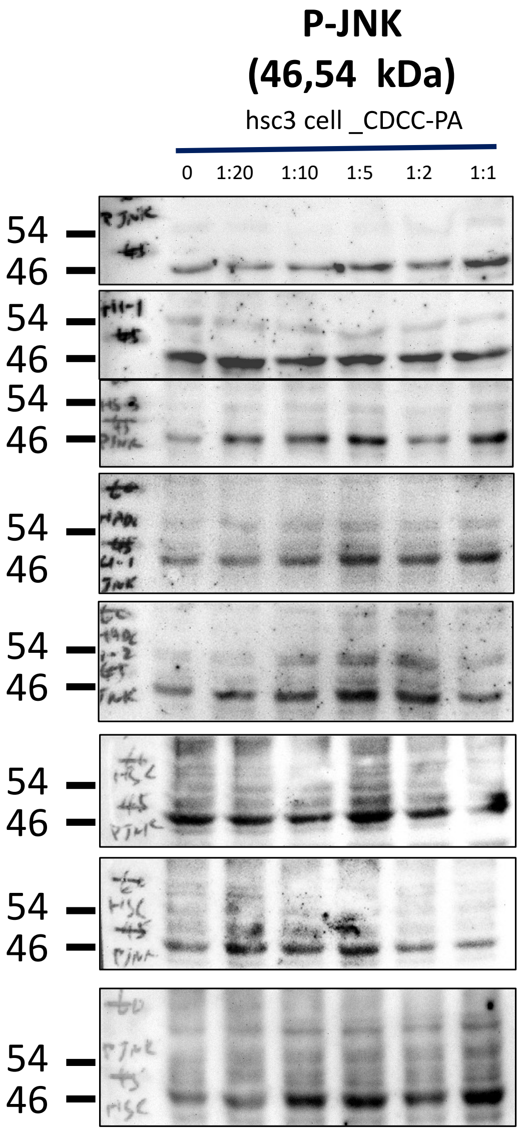

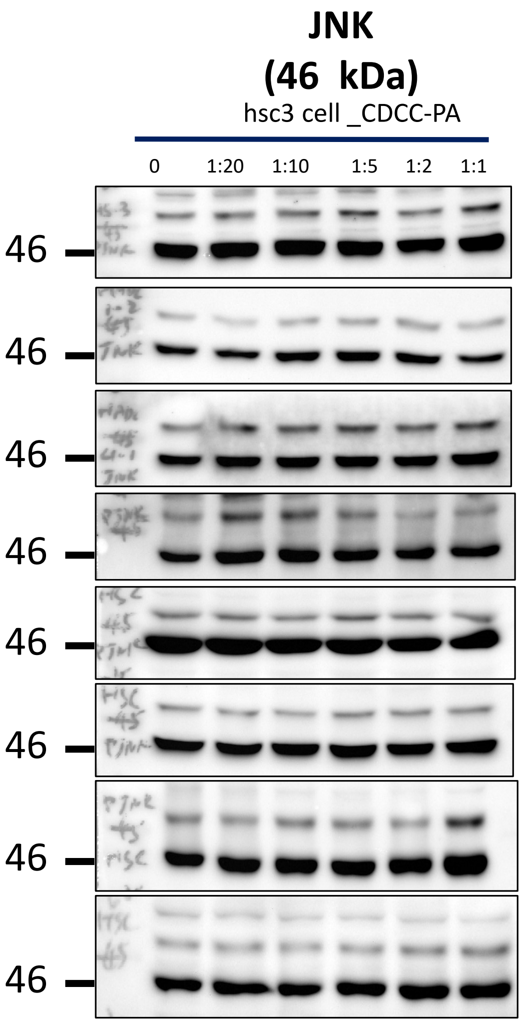

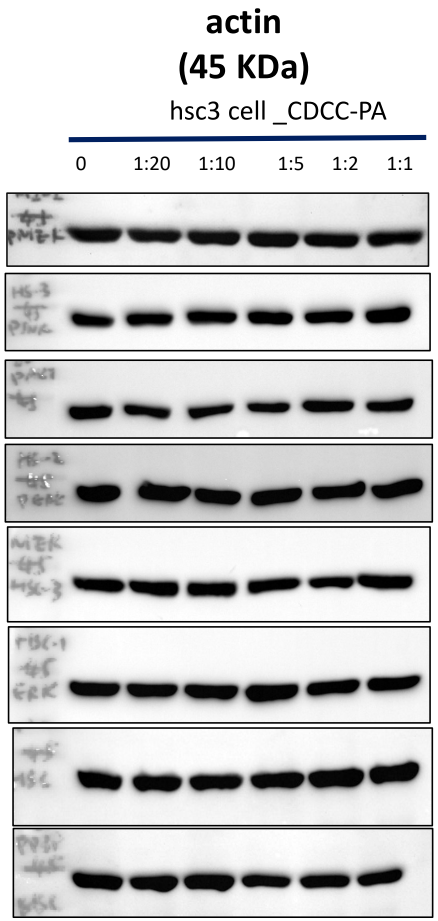

These original western blot images are related to Fig 6A in the main text.

HSC3 p-ERK, ERK (N=8)

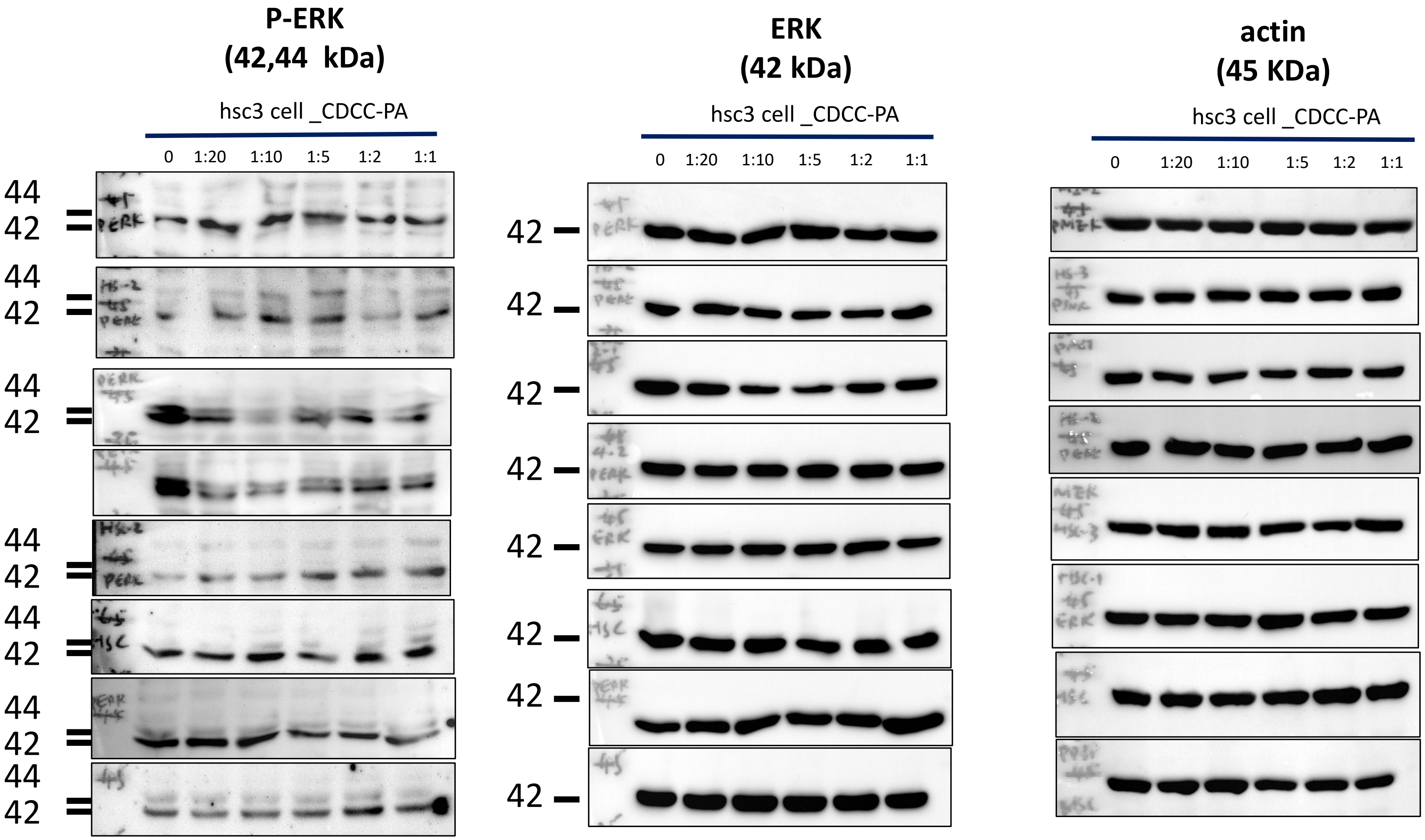

These original western blot images are related to Fig 6A in the main text.

HSC3 p-P38, P38 (N=8)

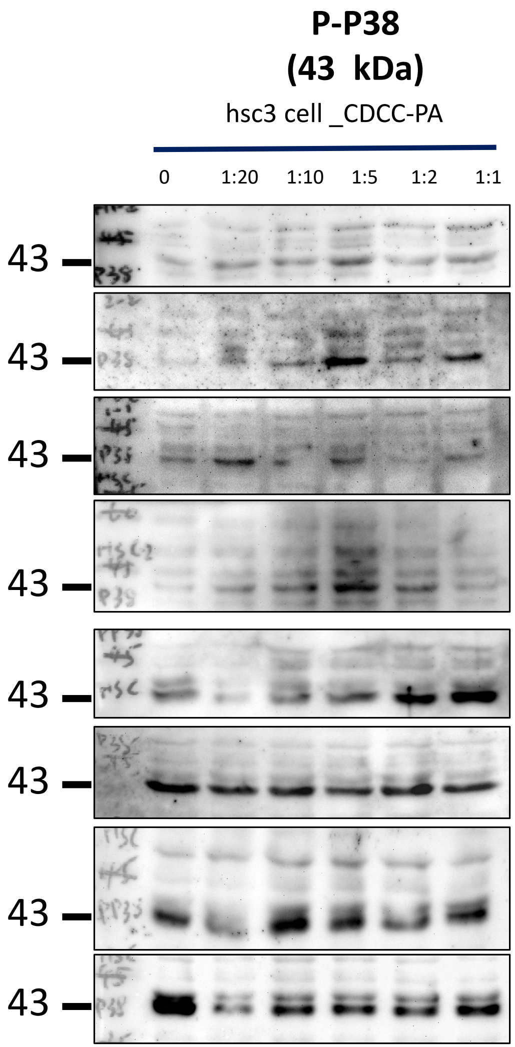

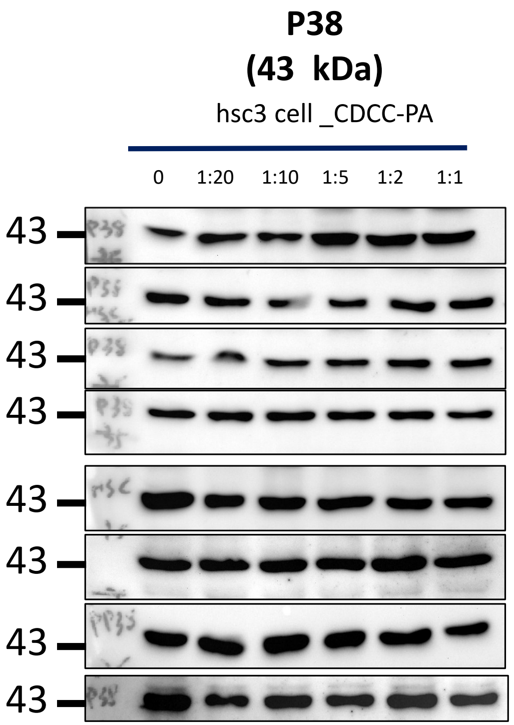

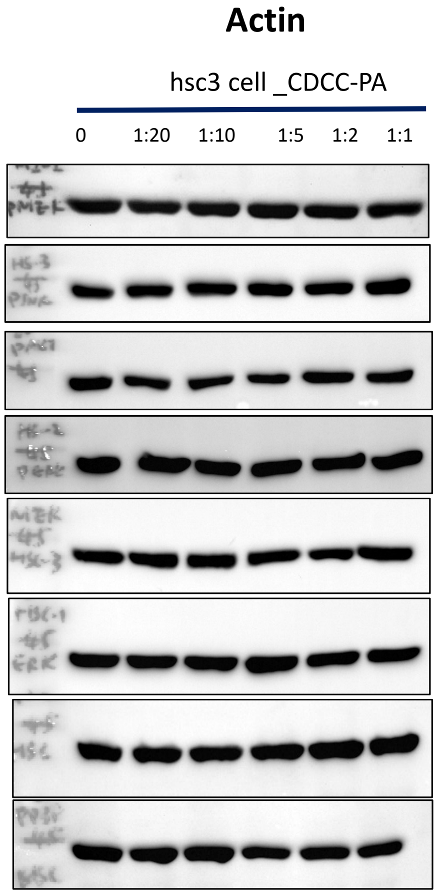

These original western blot images are related to Fig 6A in the main text.

HSC3 p-MEK, MEK (N=4)

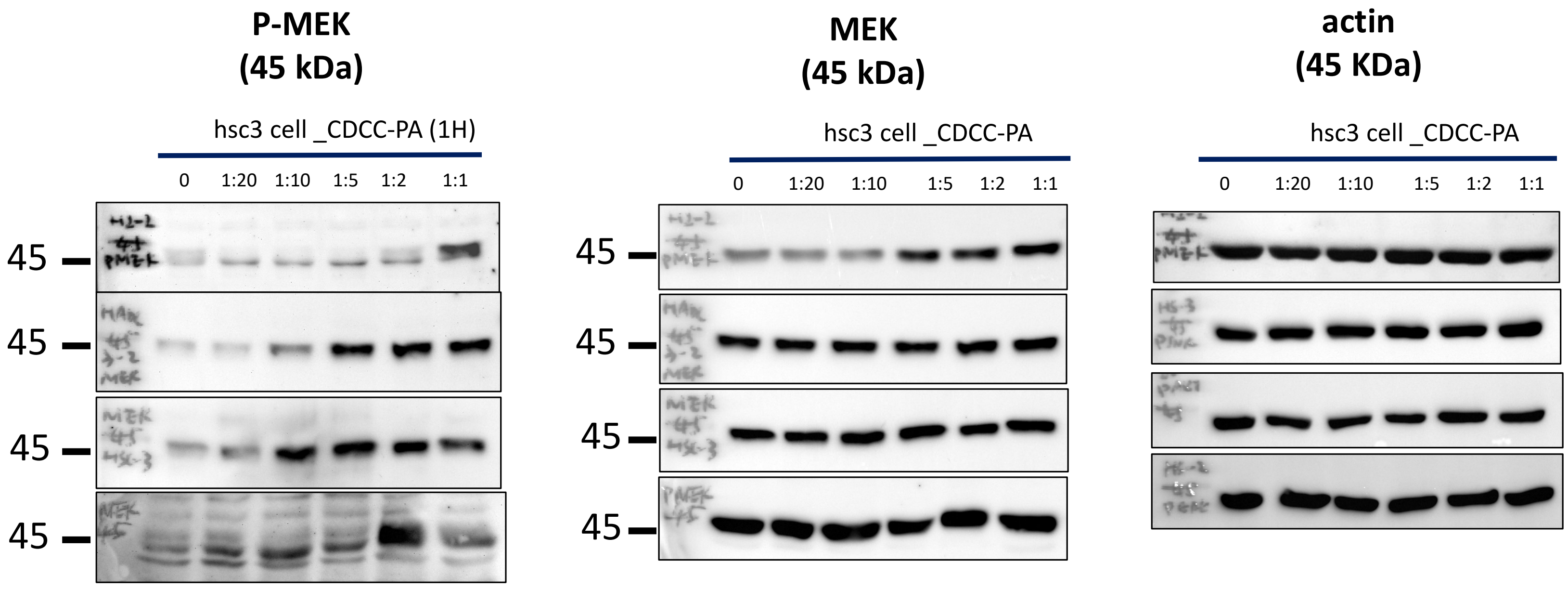

These original western blot images are related to Fig 6A in the main text.

HSC3 p-AKT, AKT (N=8)

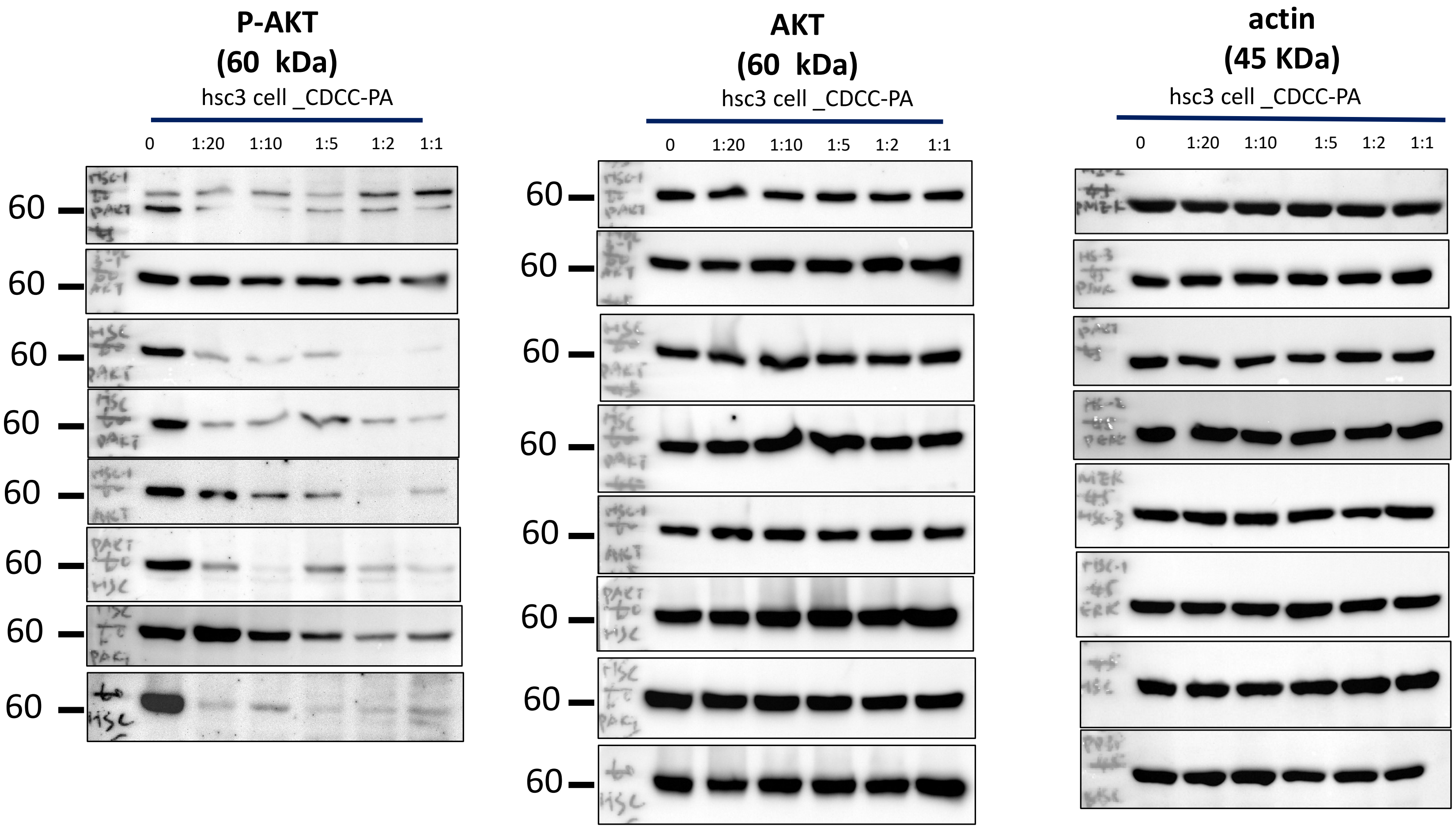

These original western blot images are related to Fig 6A in the main text.

HSC3 p-mTOR, mTOR (N=5)

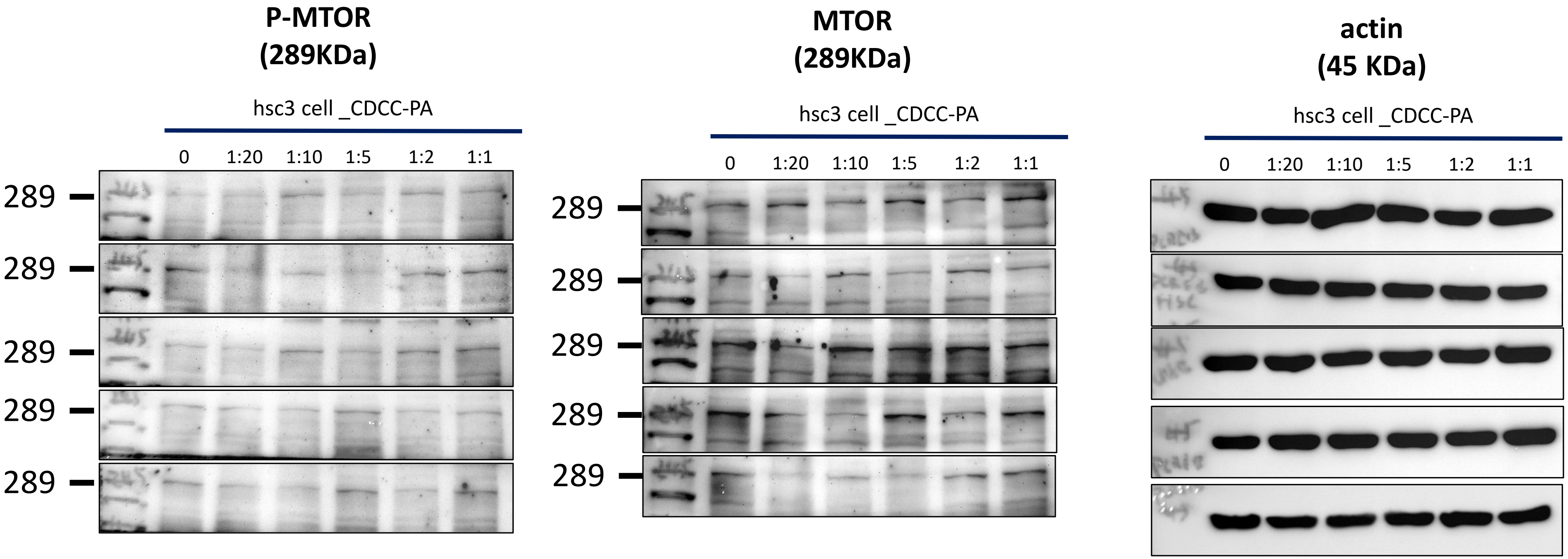

These original western blot images are related to Fig 6A in the main text.

HSC3 p-P85, P85 (N=6)

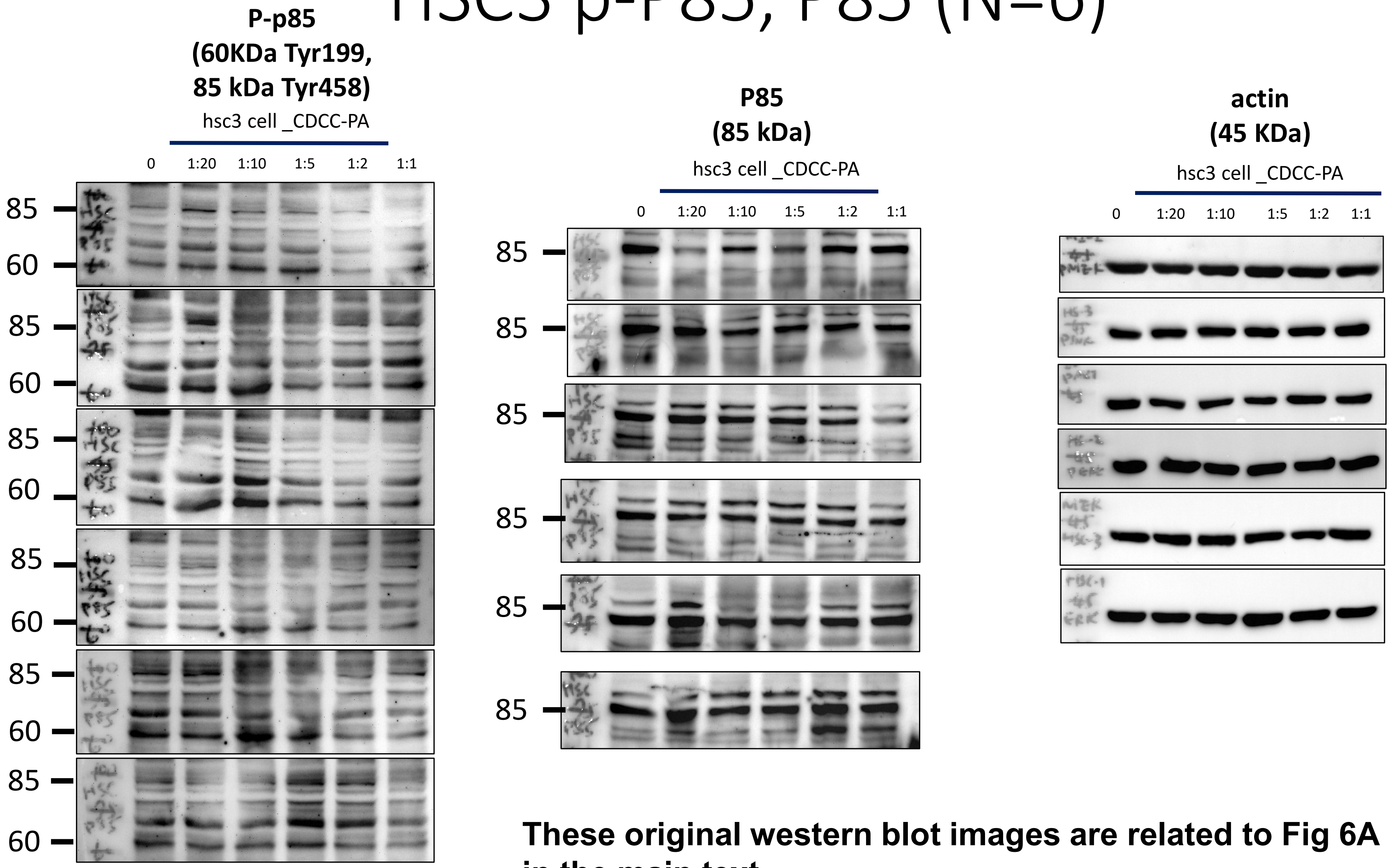

These original western blot images are related to Fig 6A in the main text.

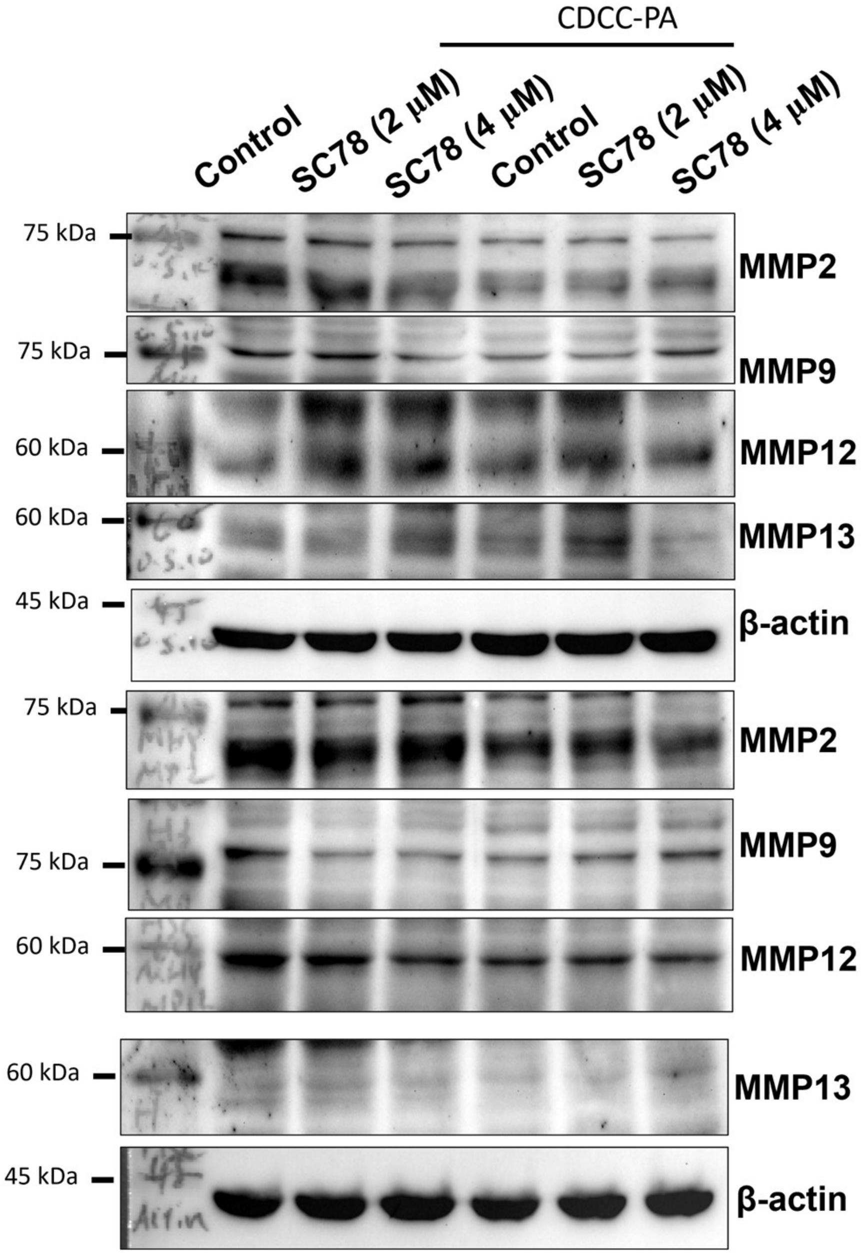

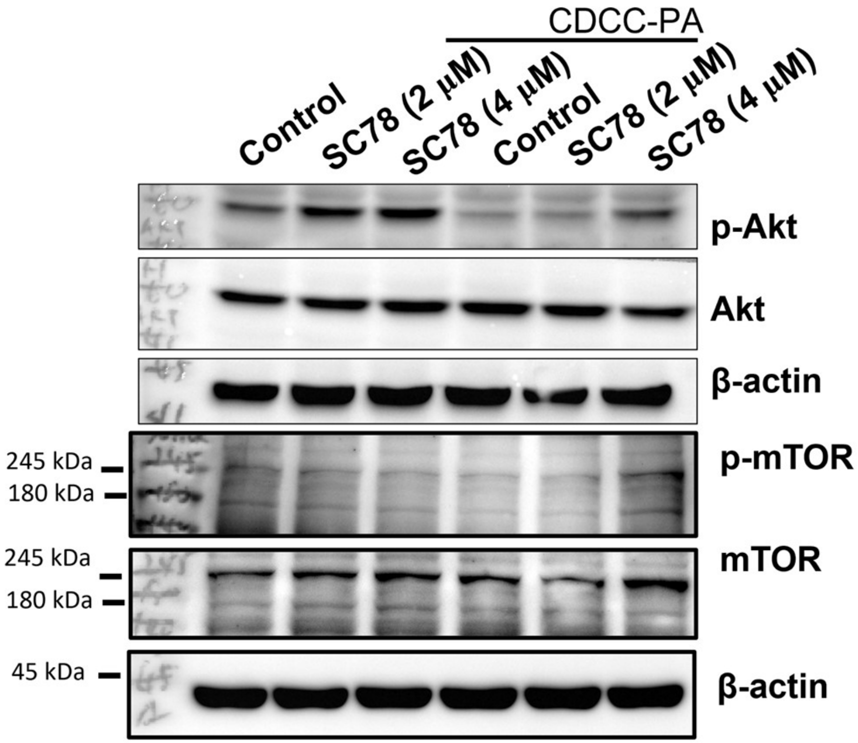

These original western blot images are related to Fig 7 in the main text.

Western blot images of SCC4 cells related experiments

These original western blot images are related to Fig 5D in the main text.

SCC4-MMP2 (N=6)

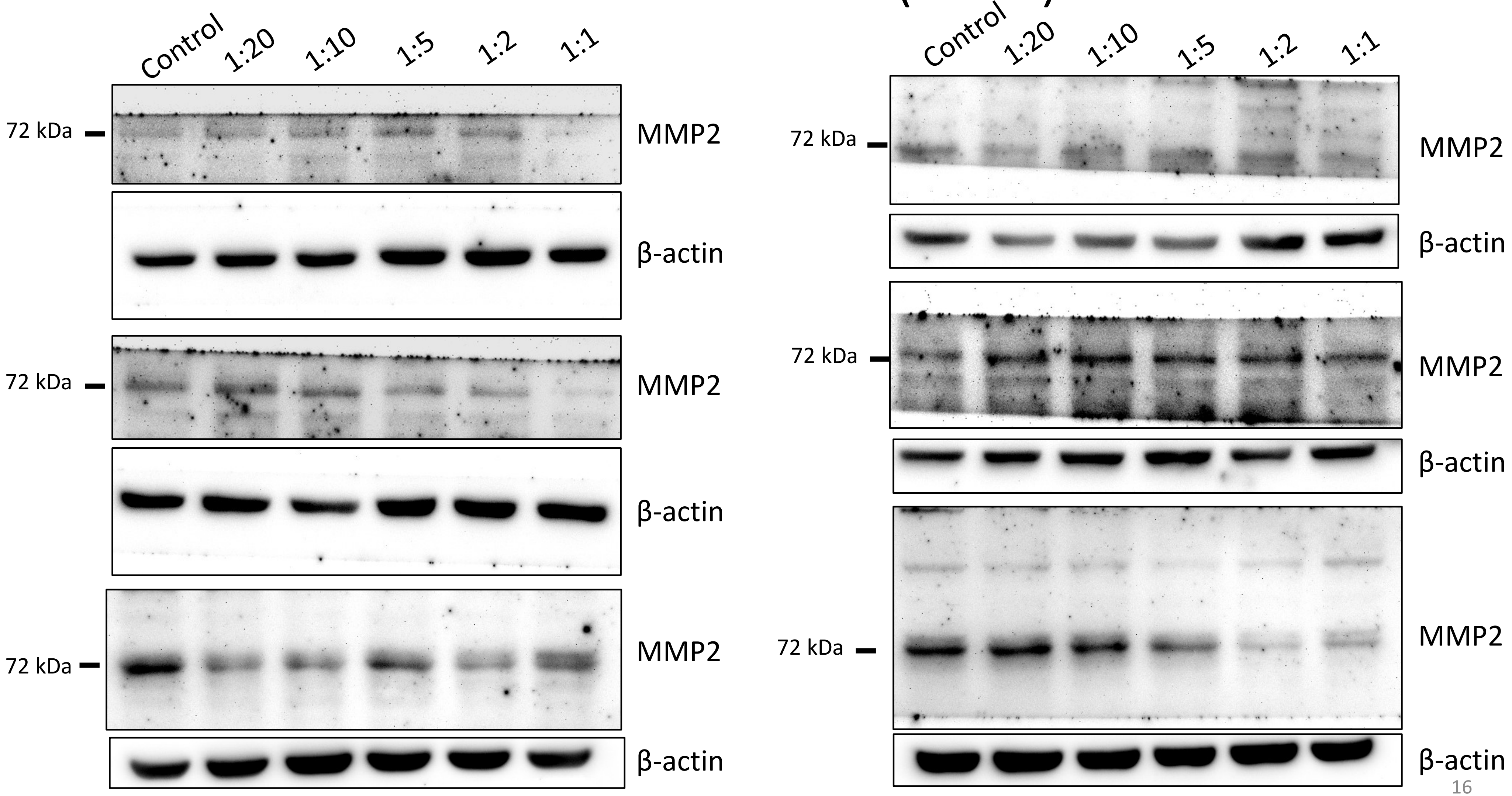

These original western blot images are related to Fig 5D in the main text.

SCC4-MMP9 (N=6)

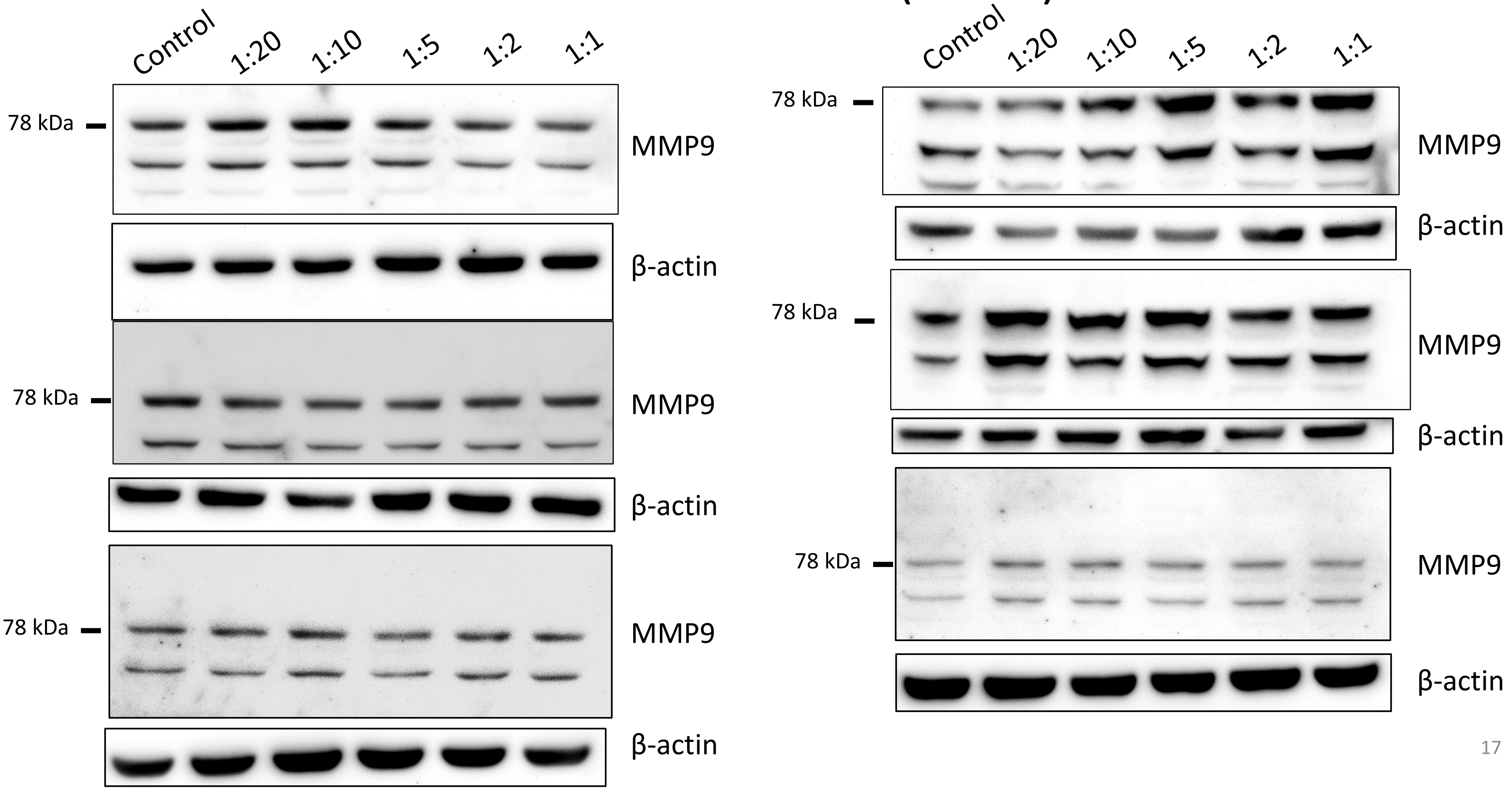

These original western blot images are related to Fig 5D in the main text.

SCC4-MMP12 (N=6)

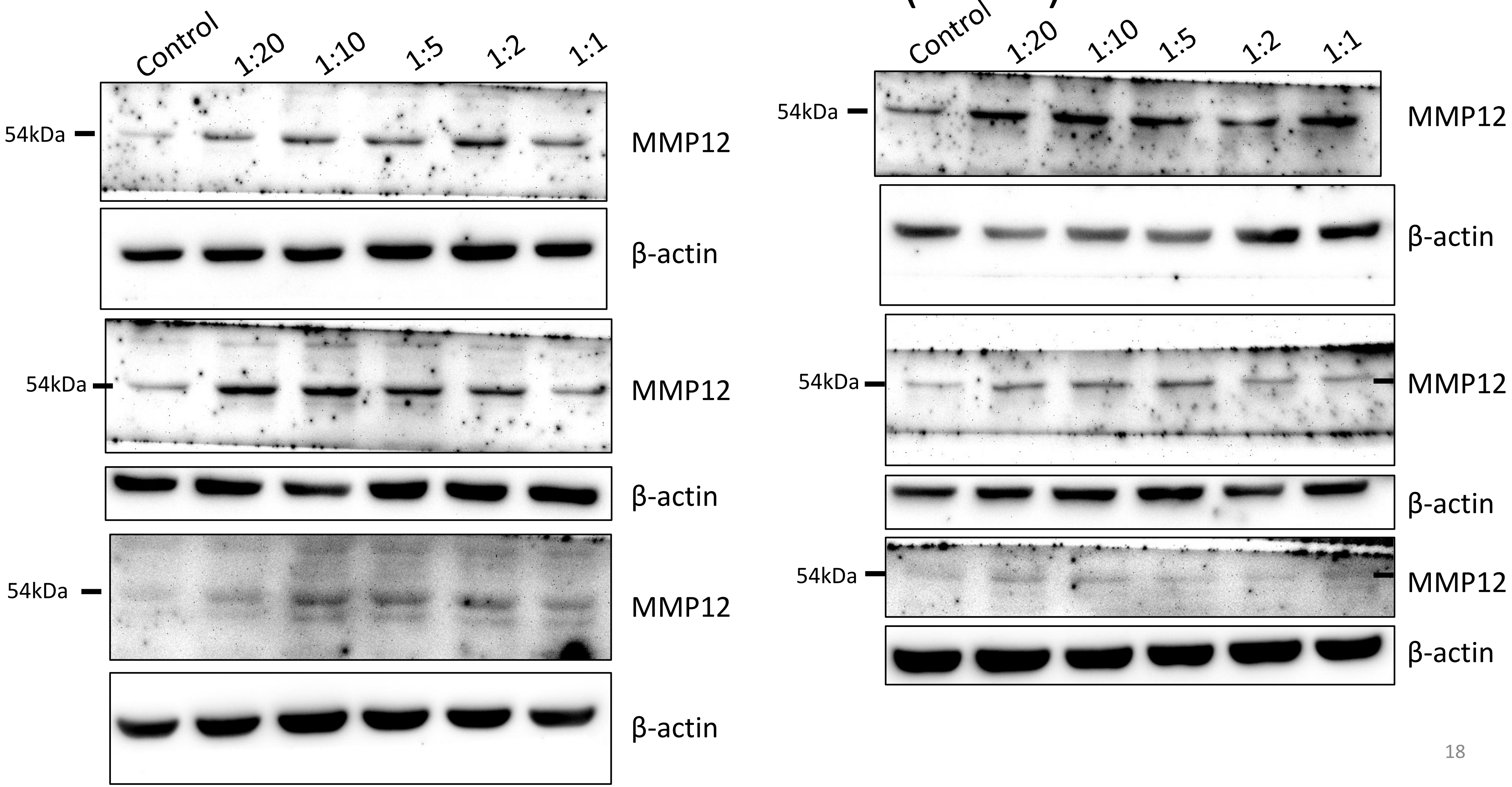

These original western blot images are related to Fig 5D in the main text.

SCC4-MMP13 (N=6)

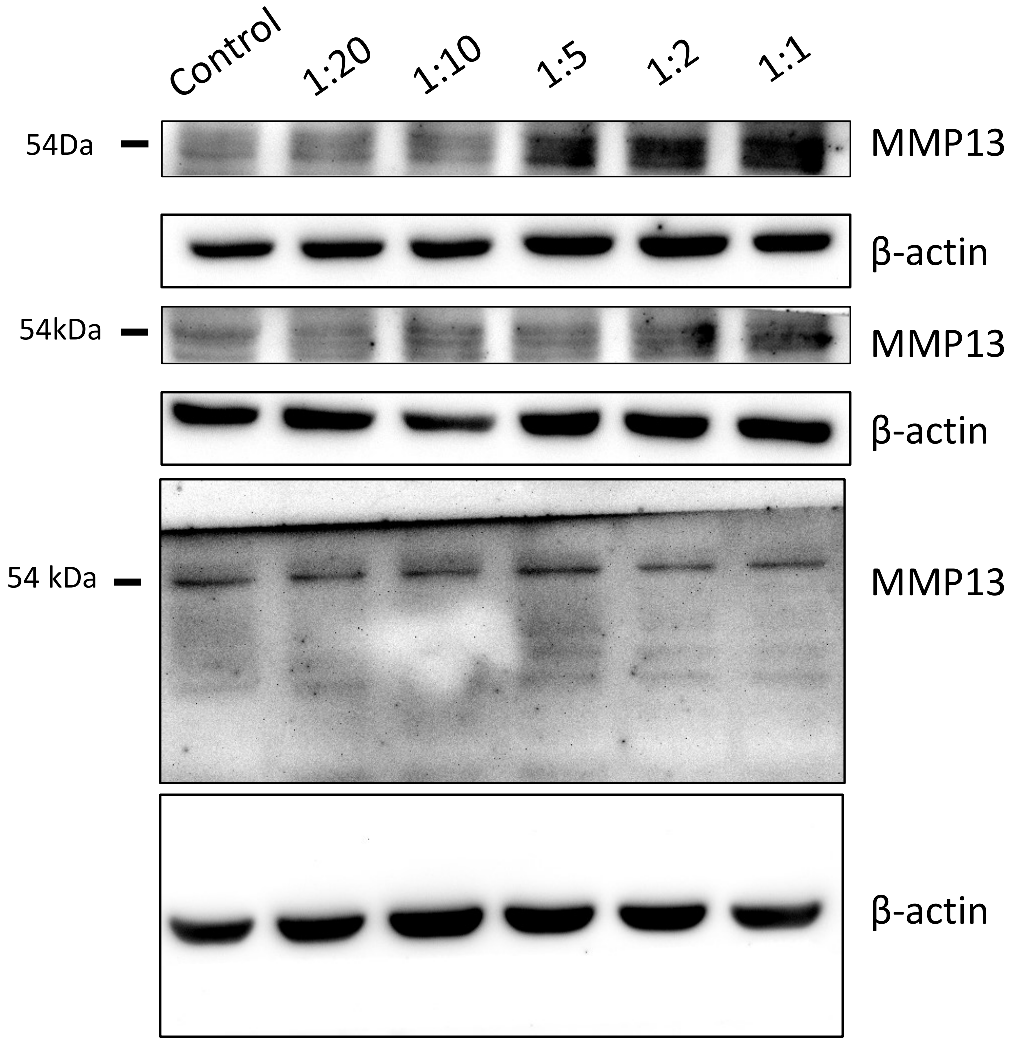

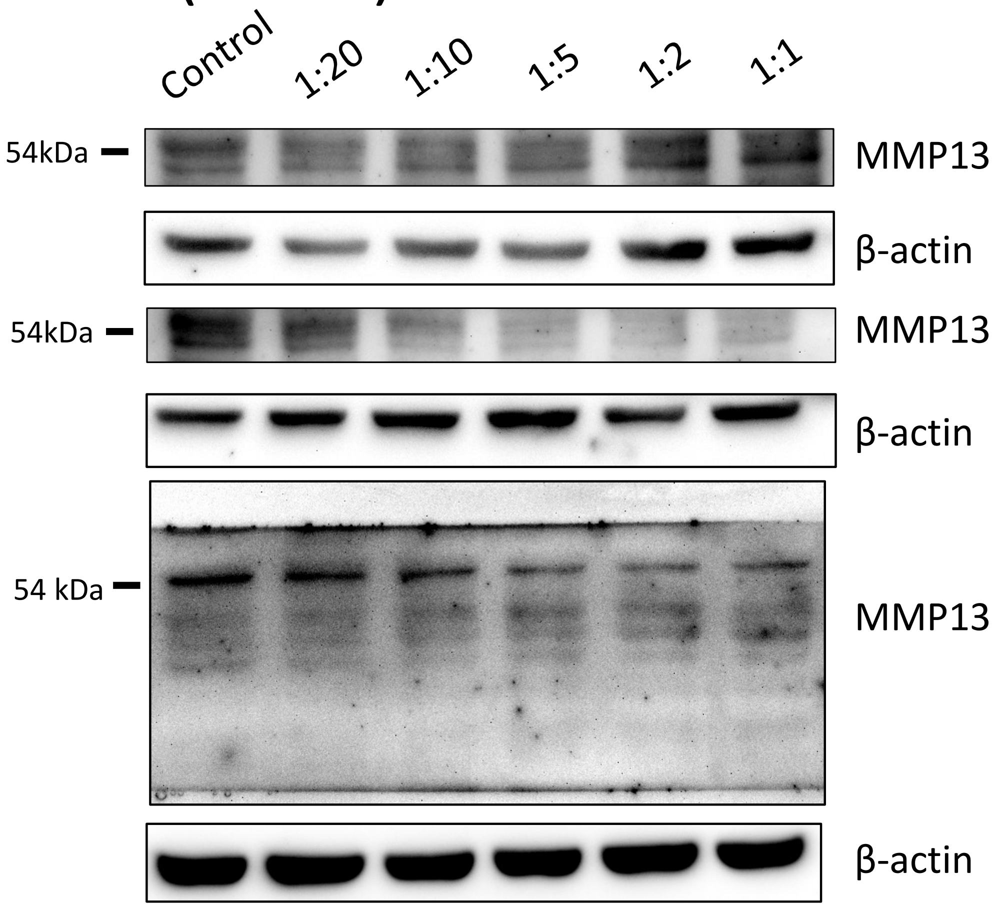

SCC4 p-P85, P85 (N=6)

P-p85 (60KDa Tyr199, 85 kDa Tyr458)

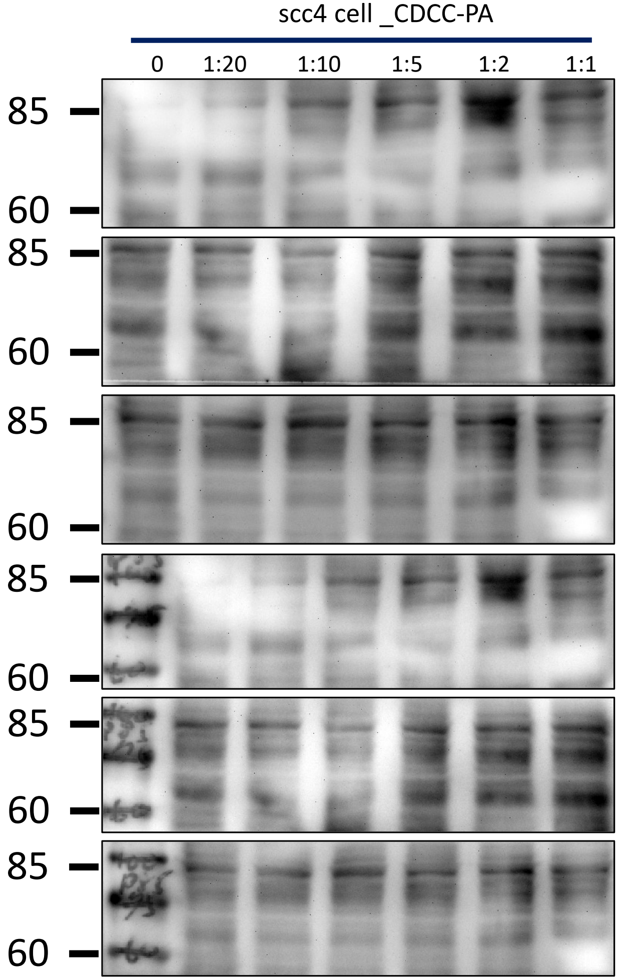

p85 (85 kDa)

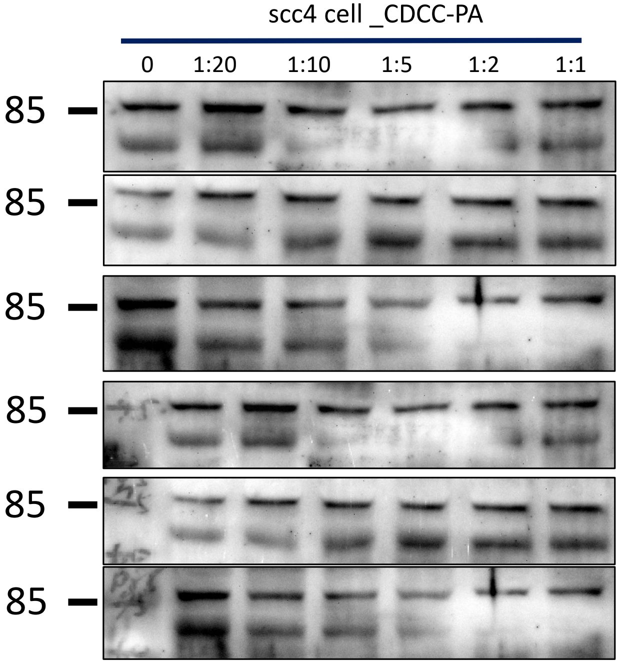

actin (45 KDa)

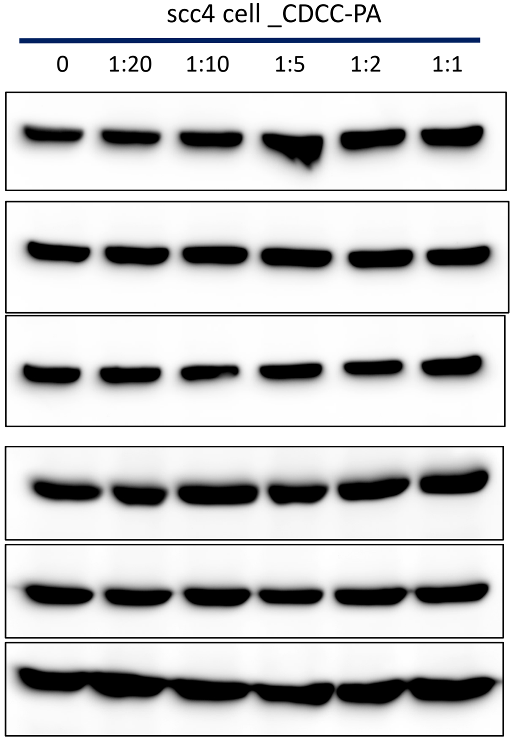

These original western blot images are related to Fig 6B in the main text.

SCC4 p-AKT, AKT (N=6)

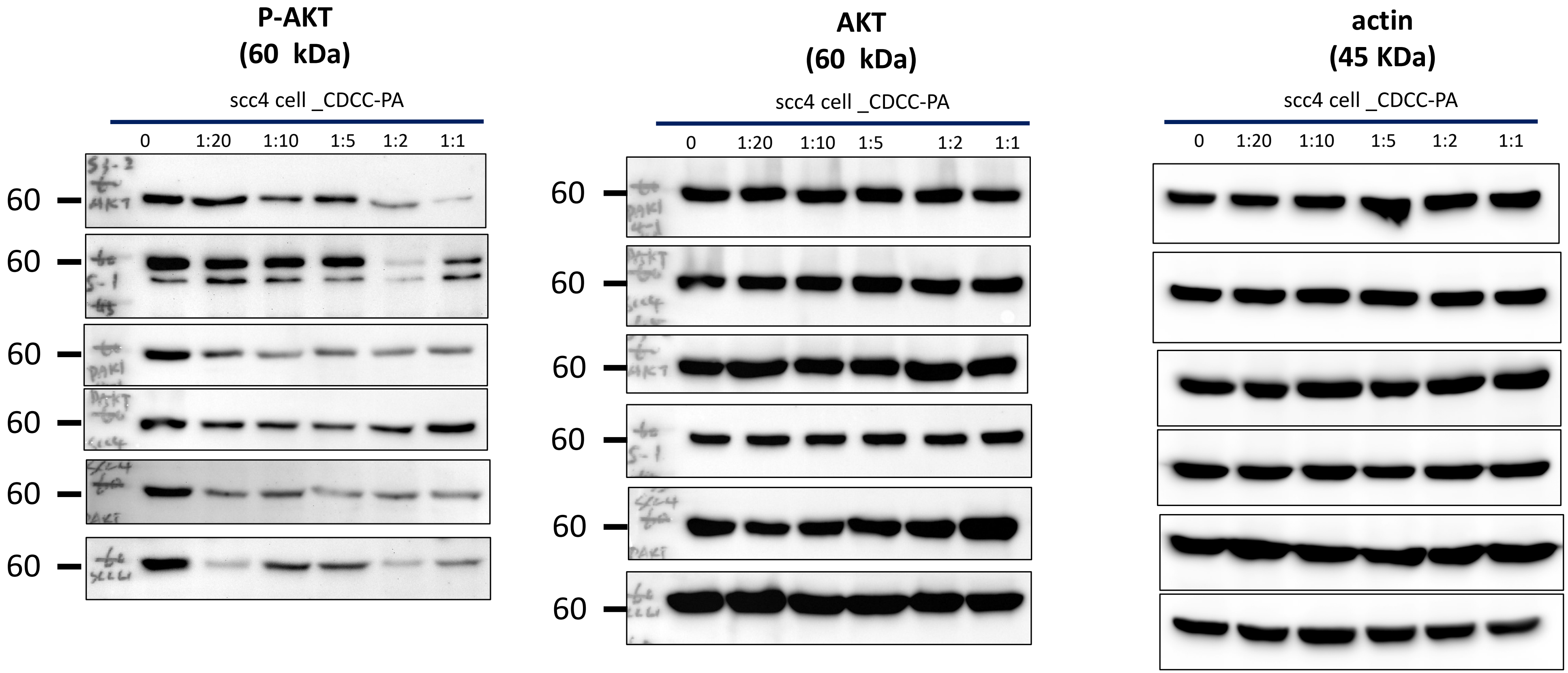

These original western blot images are related to Fig 6B in the main text.

SCC4 p-ERK, ERK (N=6)

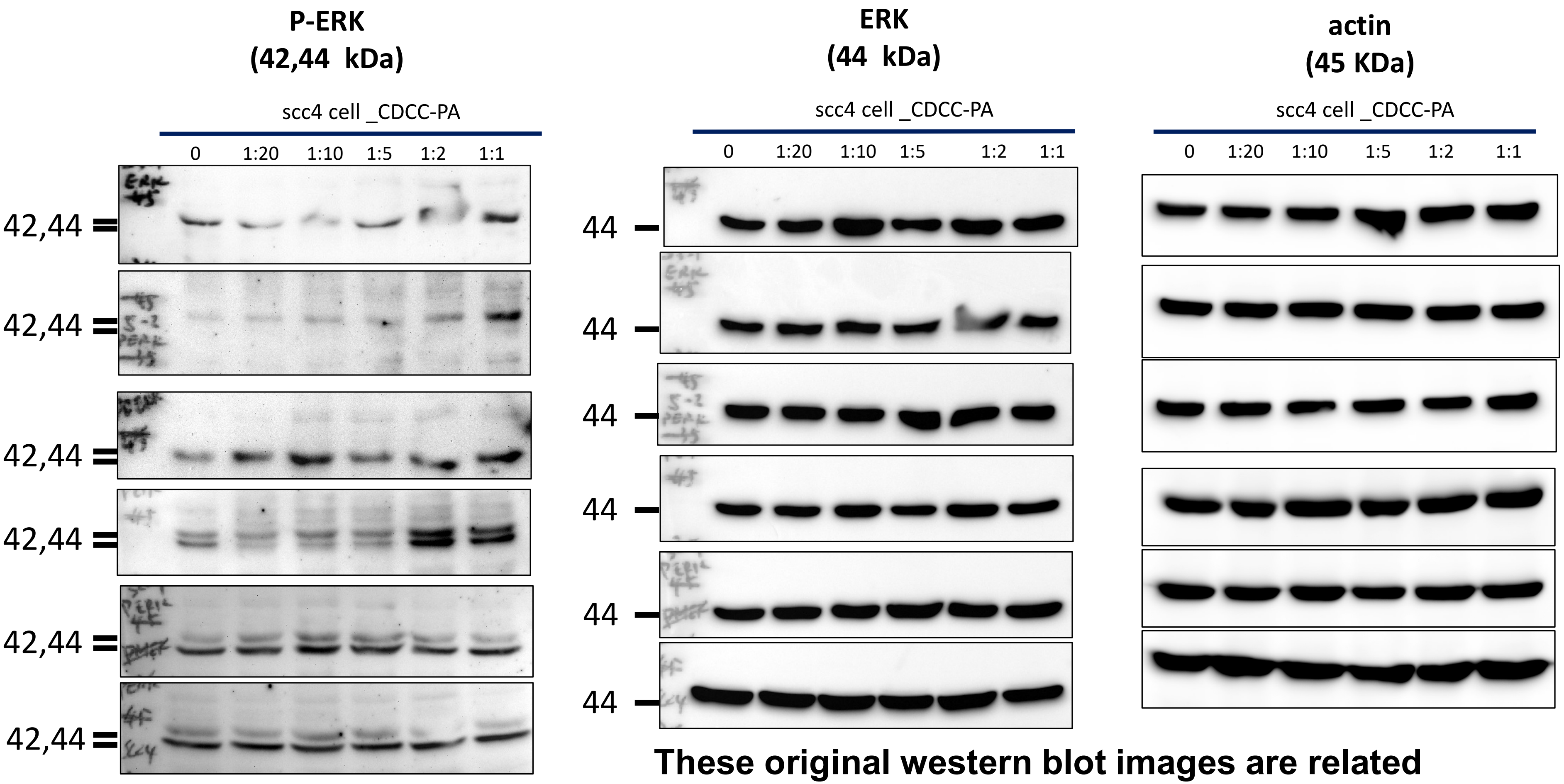

These original western blot images are related to Fig 6B in the main text.

SCC4 p-MEK, MEK (N=6)

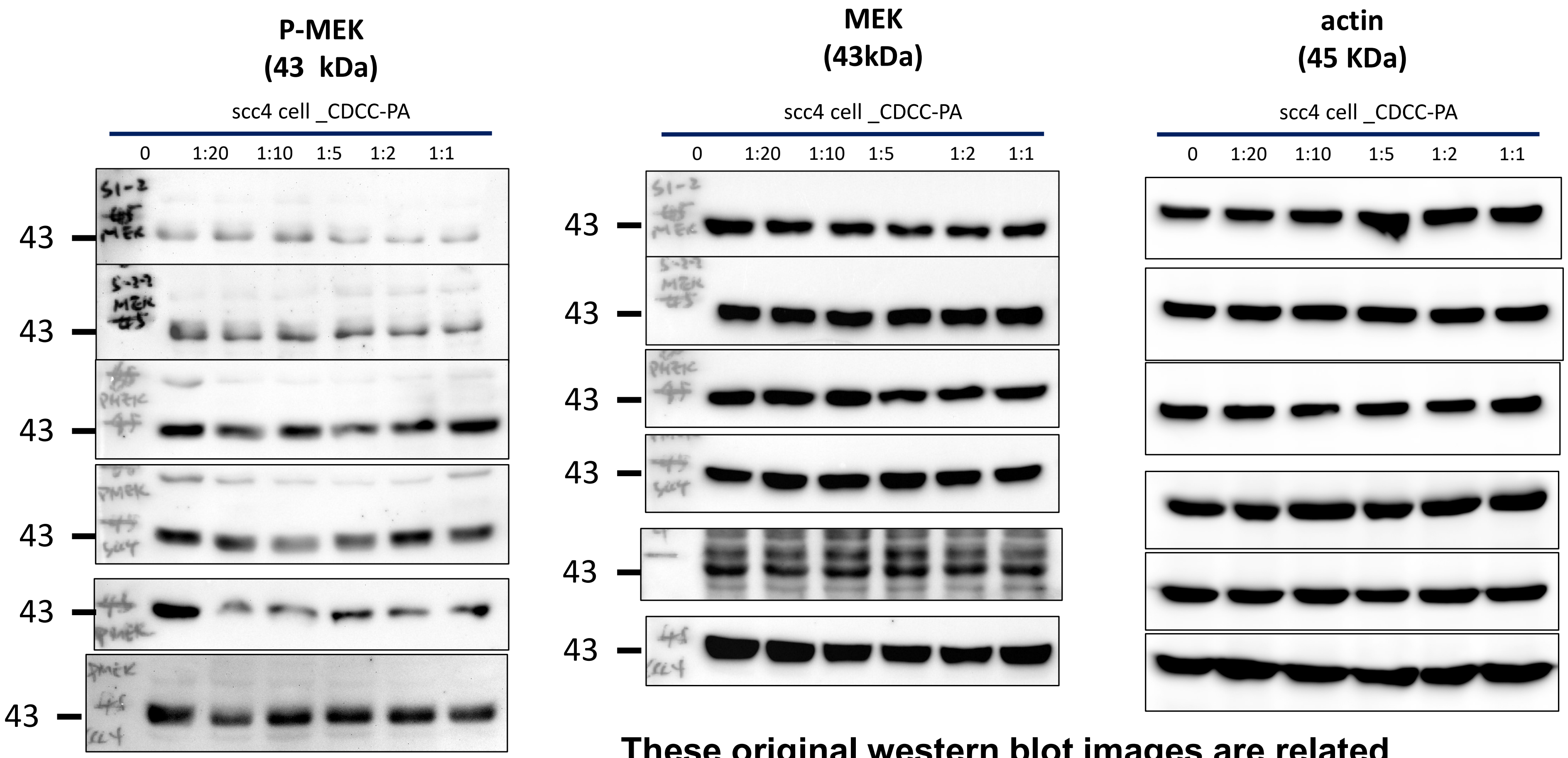

These original western blot images are related to Fig 6B in the main text.

SCC4 p-P38, P38 (N=6)

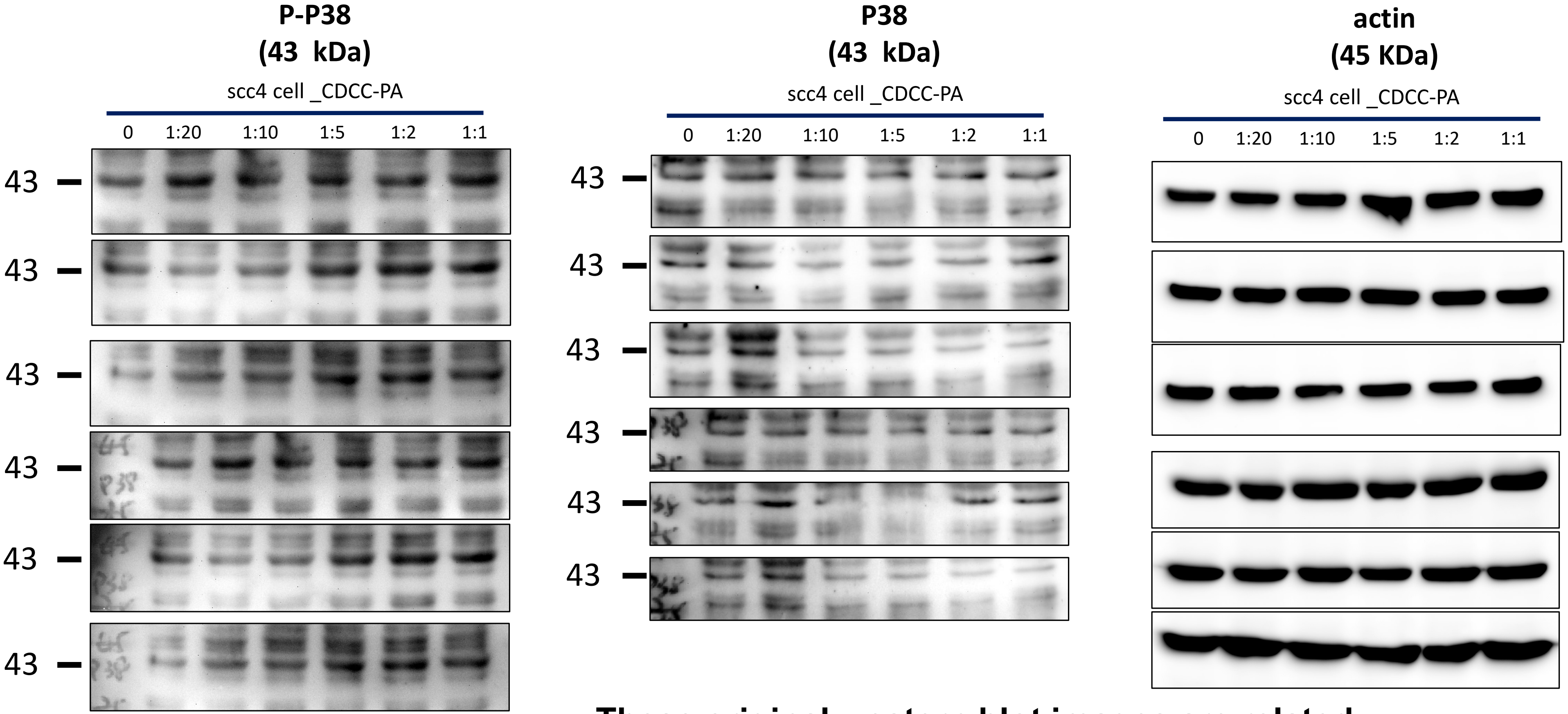

These original western blot images are related to Fig 6B in the main text.

SCC4 p-JNK, JNK

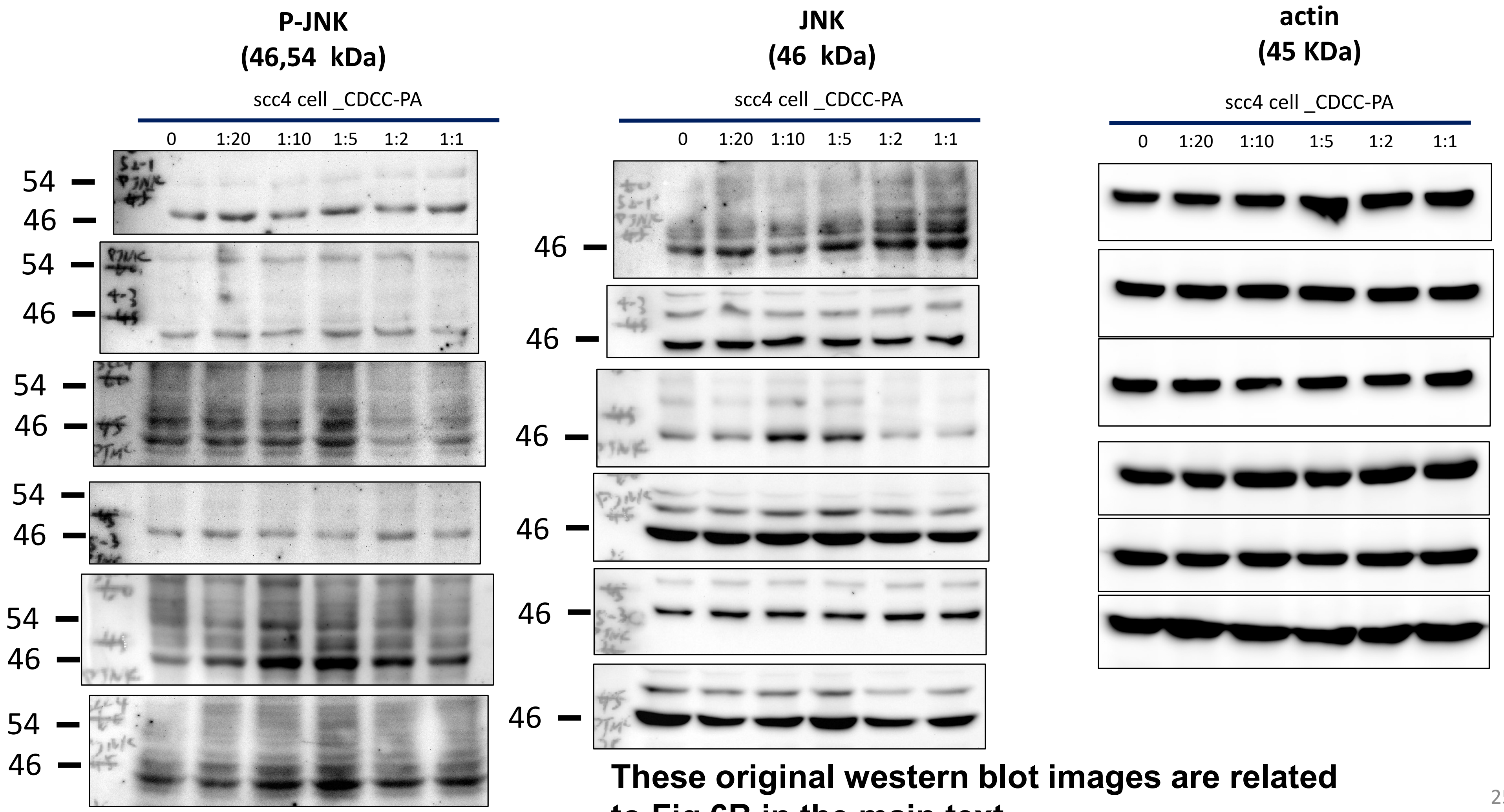

These original western blot images are related to Fig 6B in the main text.

SCC4 p-mTOR, mTOR

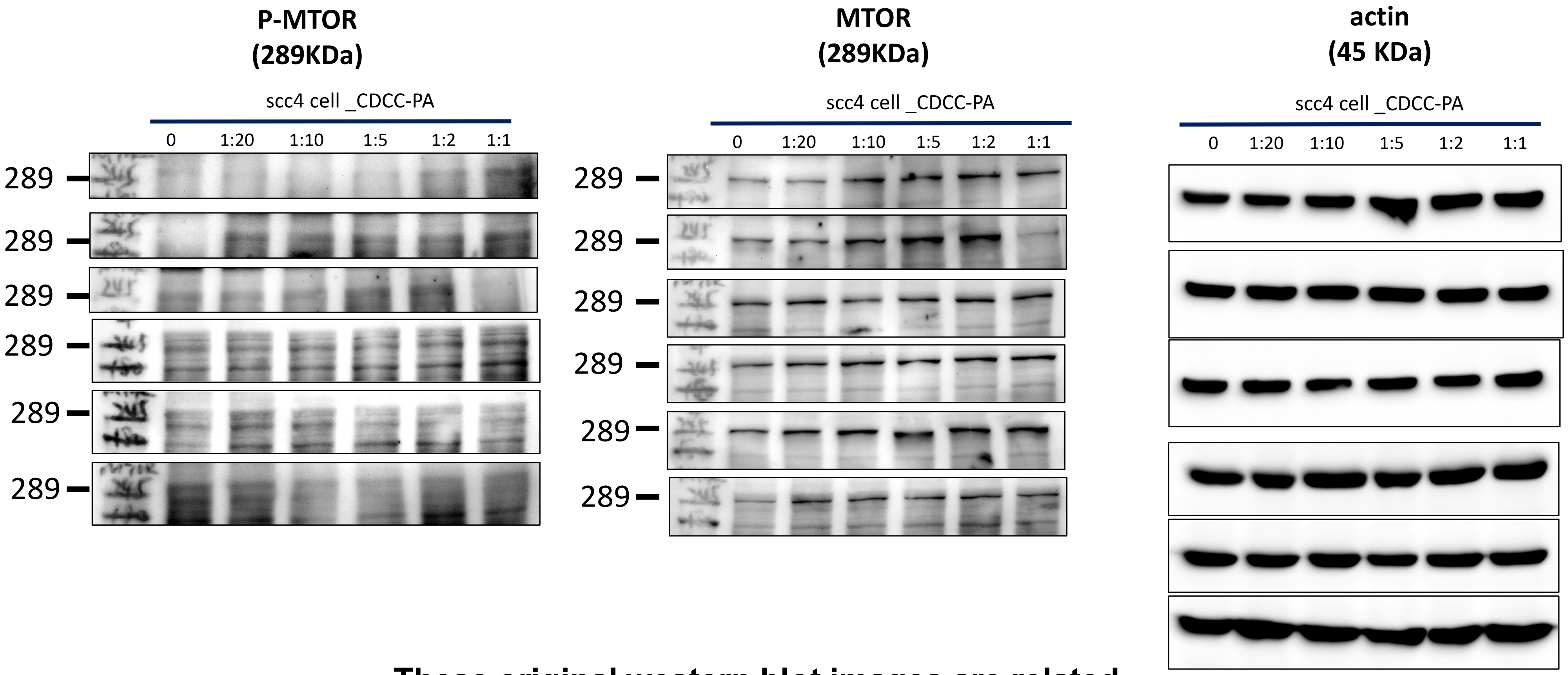

These original western blot images are related to Fig 6B in the main text.

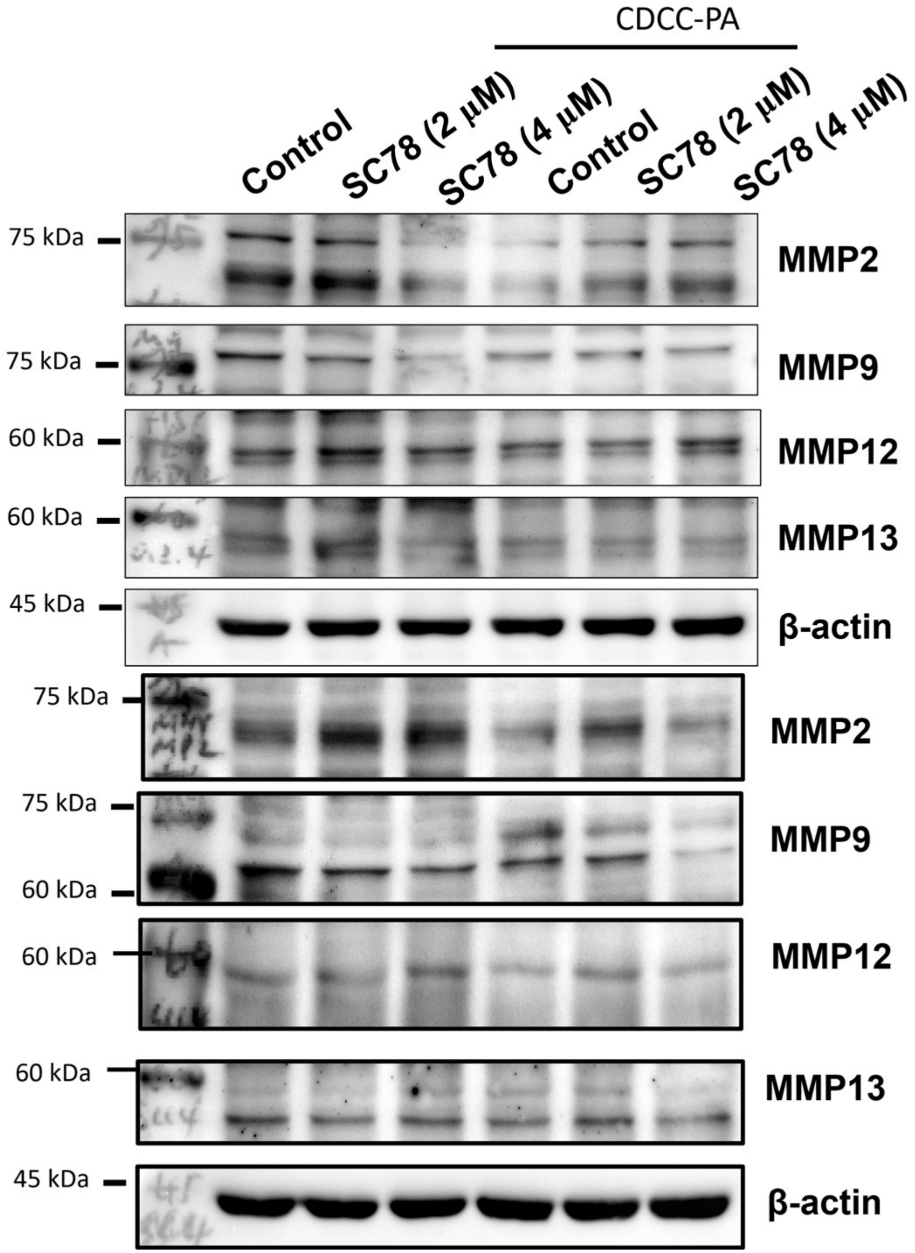

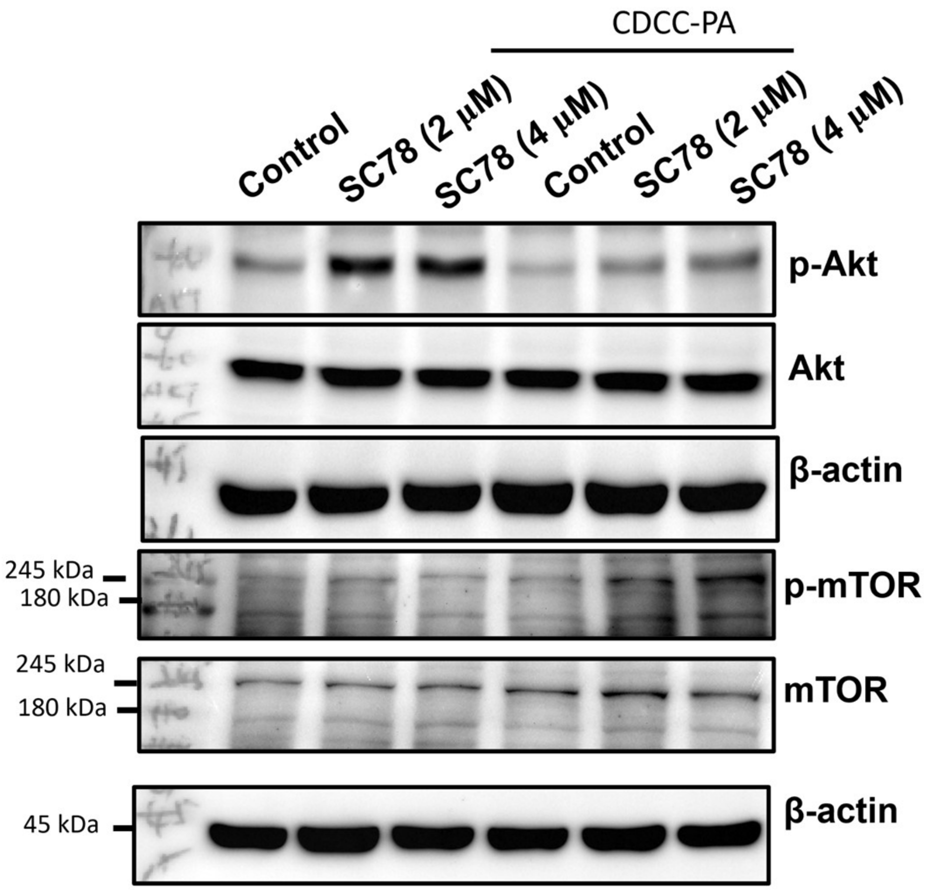

These original western blot images are related to Fig 7 in the main text.

Gel zymography Results of MMPs expression in oral cancer cell lines

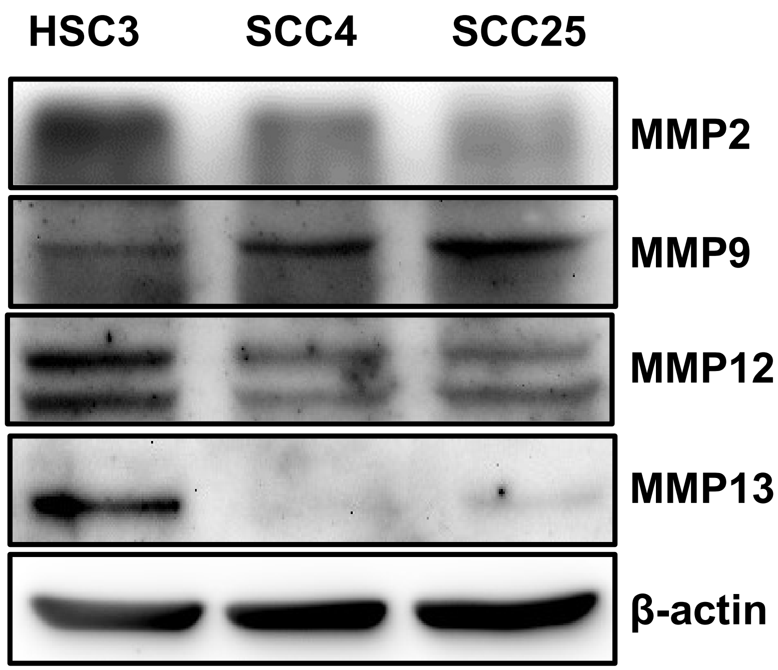
HSC3
SCC4
SCC25
MMP2
MMP9
MMP12
MMP13
β-actin

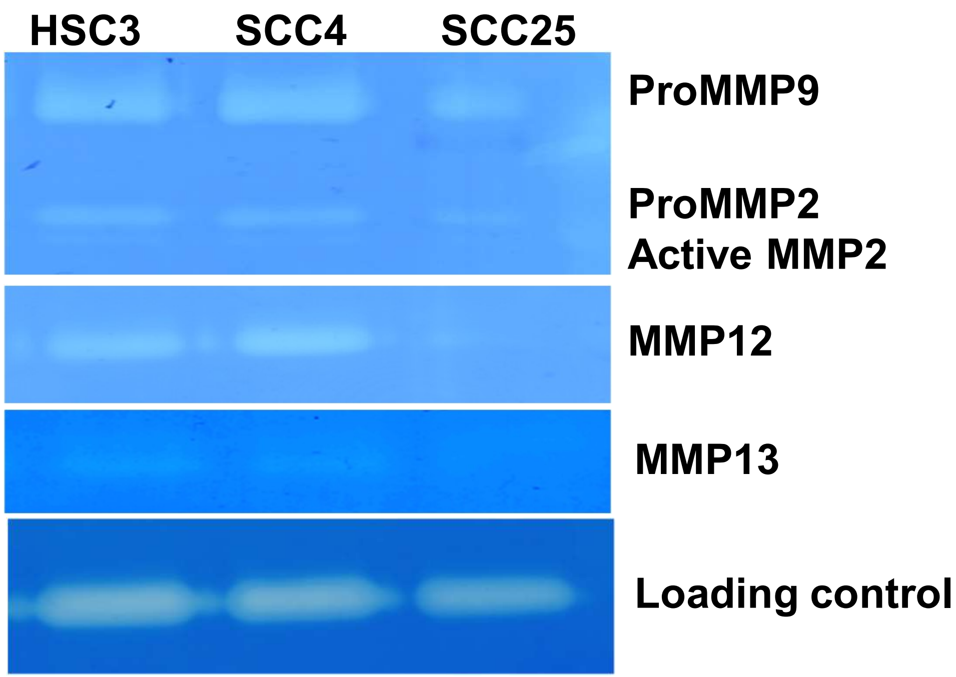
HSC3
SCC4
SCC25
ProMMP9
ProMMP2
Active MMP2
MMP12
MMP13
Loading control

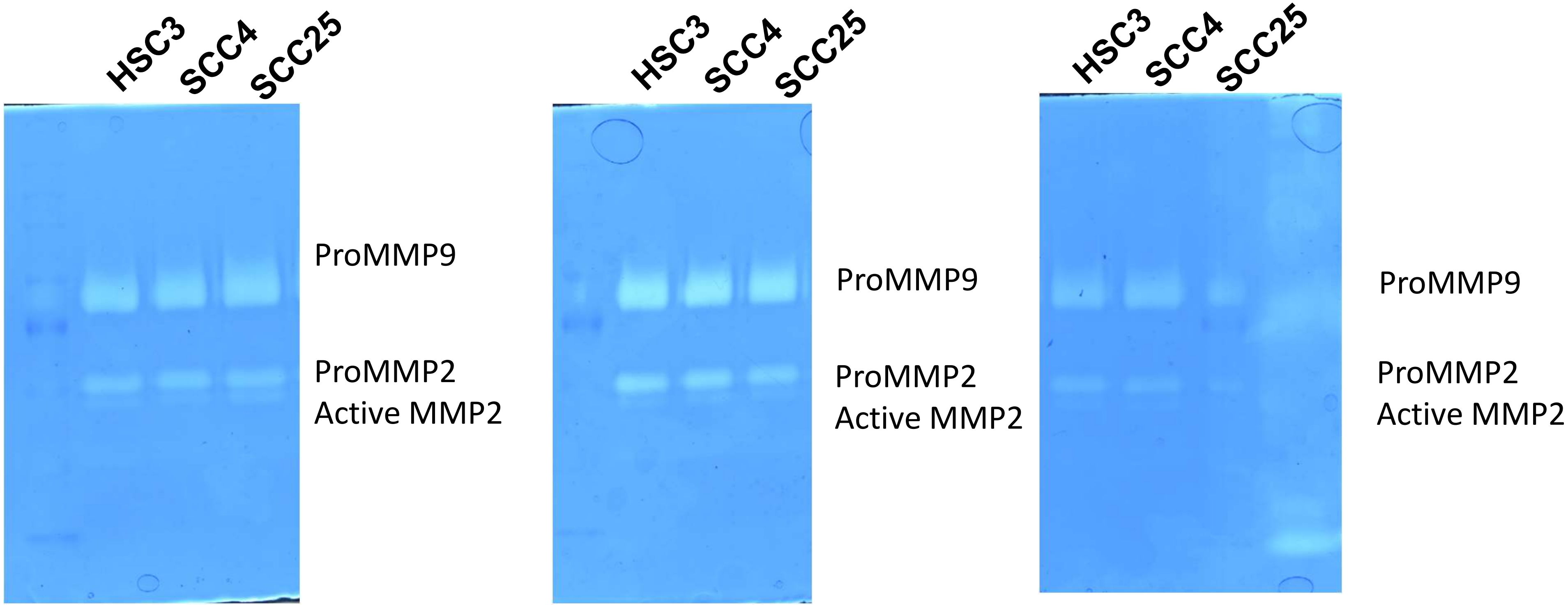
HSC3
SCC4
SCC25
ProMMP9
ProMMP2
Active MMP2
HSC3
SCC4
SCC25
ProMMP9
ProMMP2
Active MMP2
HSC3
SCC4
SCC25
ProMMP9
ProMMP2
Active MMP2

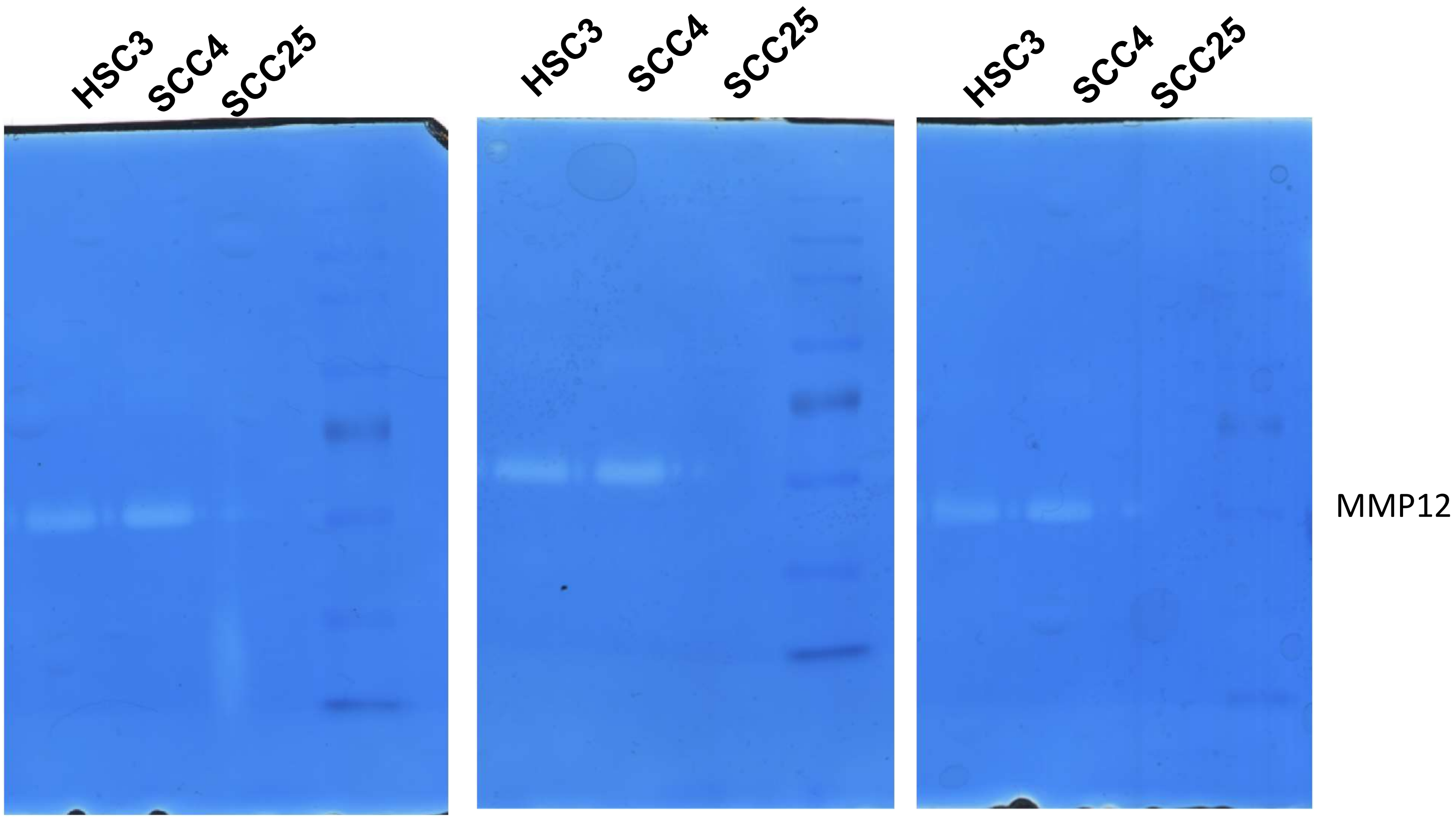
HSC3
SCC4
SCC25
HSC3
SCC4
SCC25
HSC3
SCC4
SCC25
MMP12

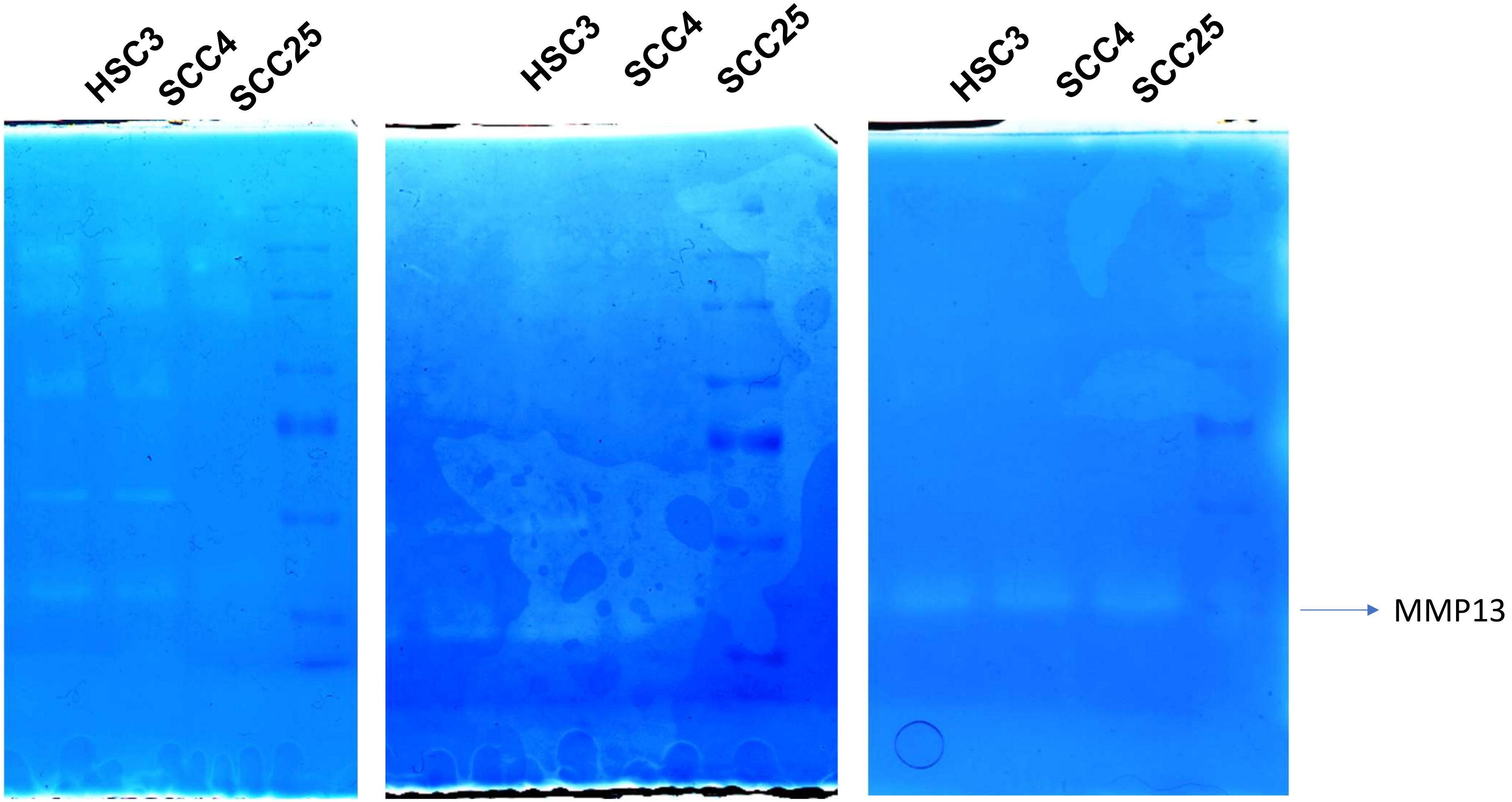
HSC3
SCC4
SCC25
HSC3
SCC4
SCC25
HSC3
SCC4
SCC25
MMP13

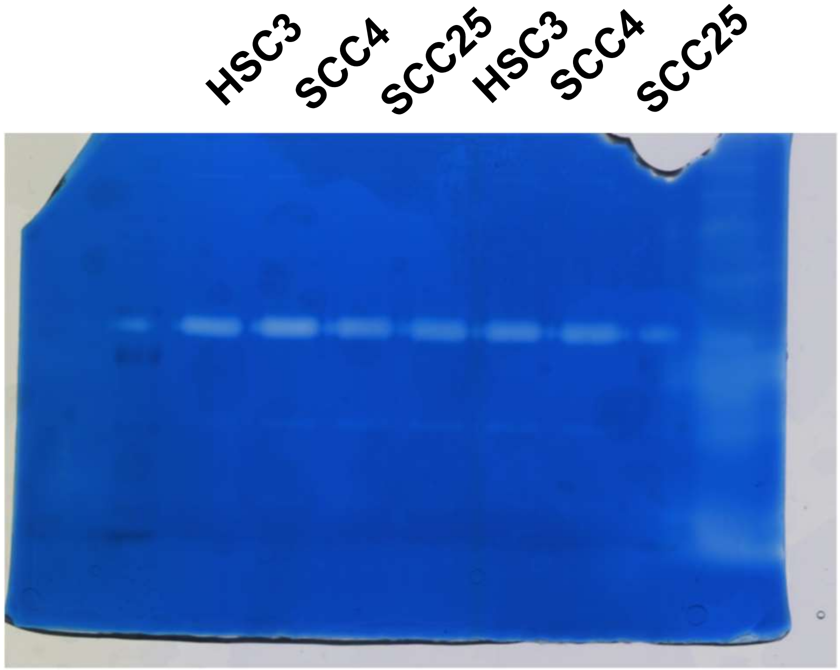

Loading control : 25µl culture medium

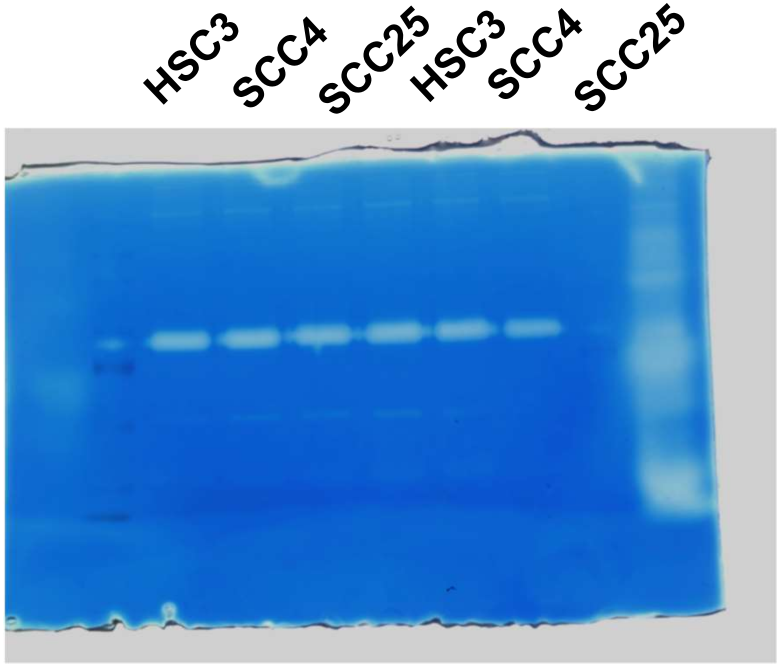

Loading control : 25µl culture medium

To examine whether CCDC-PA stimulates the production of MMPs through Akt signaling, Transfecting cells with Akt dominant mutants (DN-Akt) enhanced CCDC-PA-inhibited MMPs expression ngative Akt mutant to determine the role of MMP under the influence of CCDC-PA

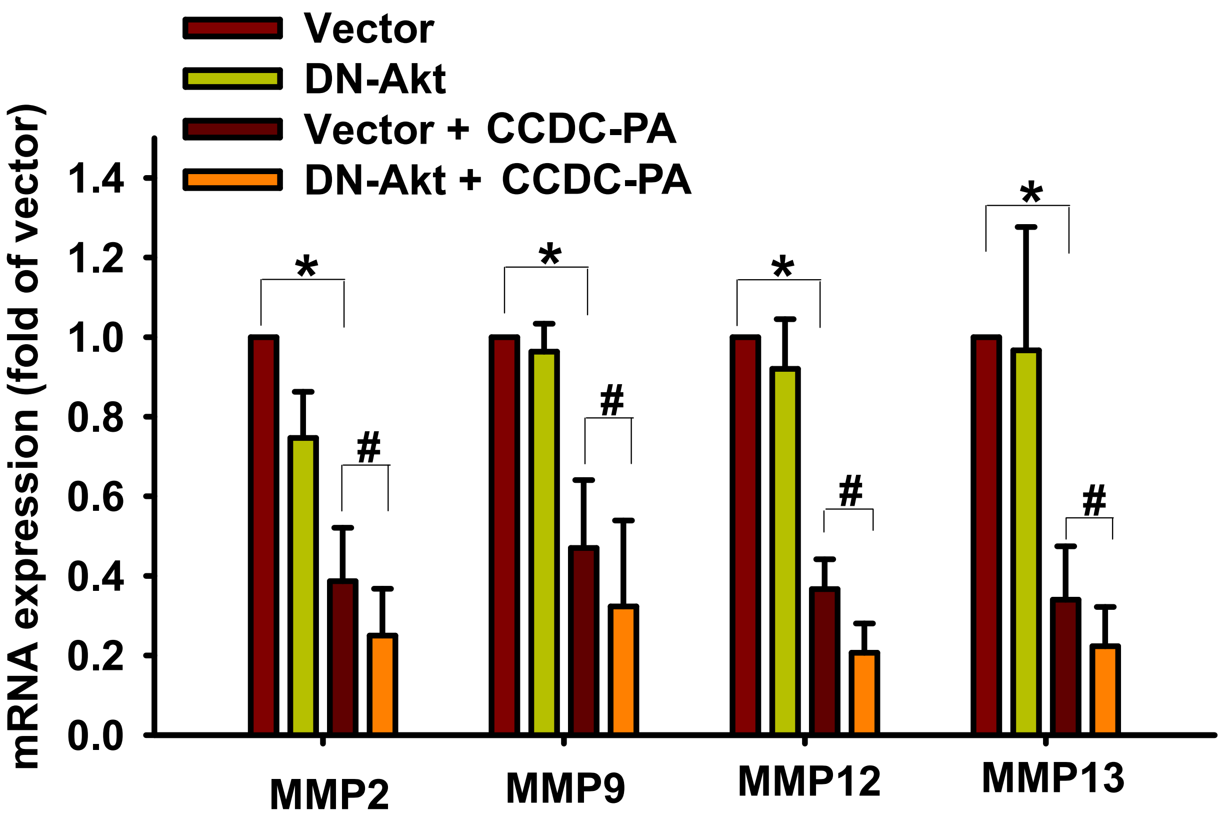

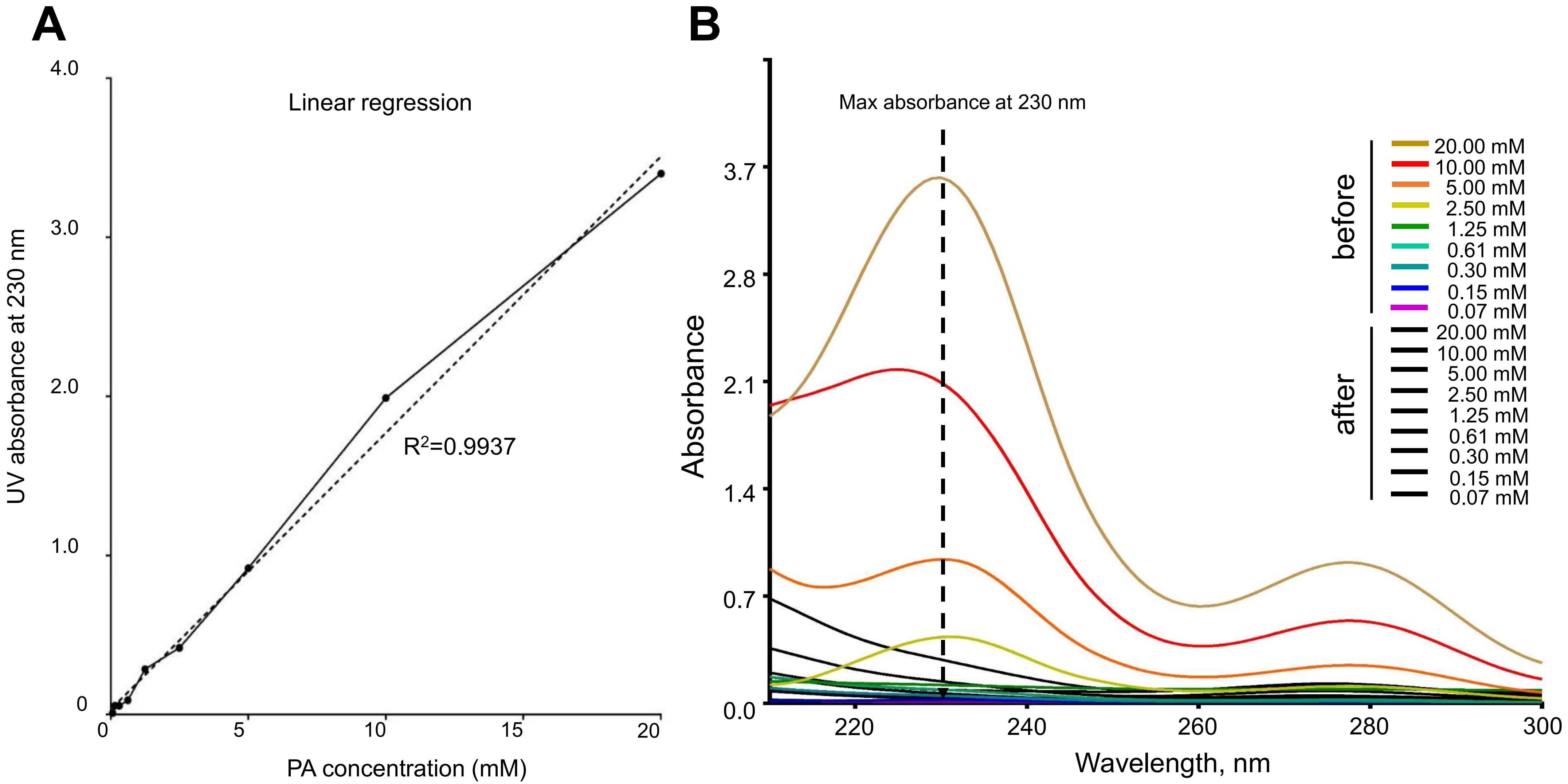

The encapsulation capacity of proanthocyanidins (PA) into the ADC system. (A) UV absorbance intensity as a function of PA concentration in solution. (B) The UV–Vis spectra of PA solution before and after the loading into ADC system.

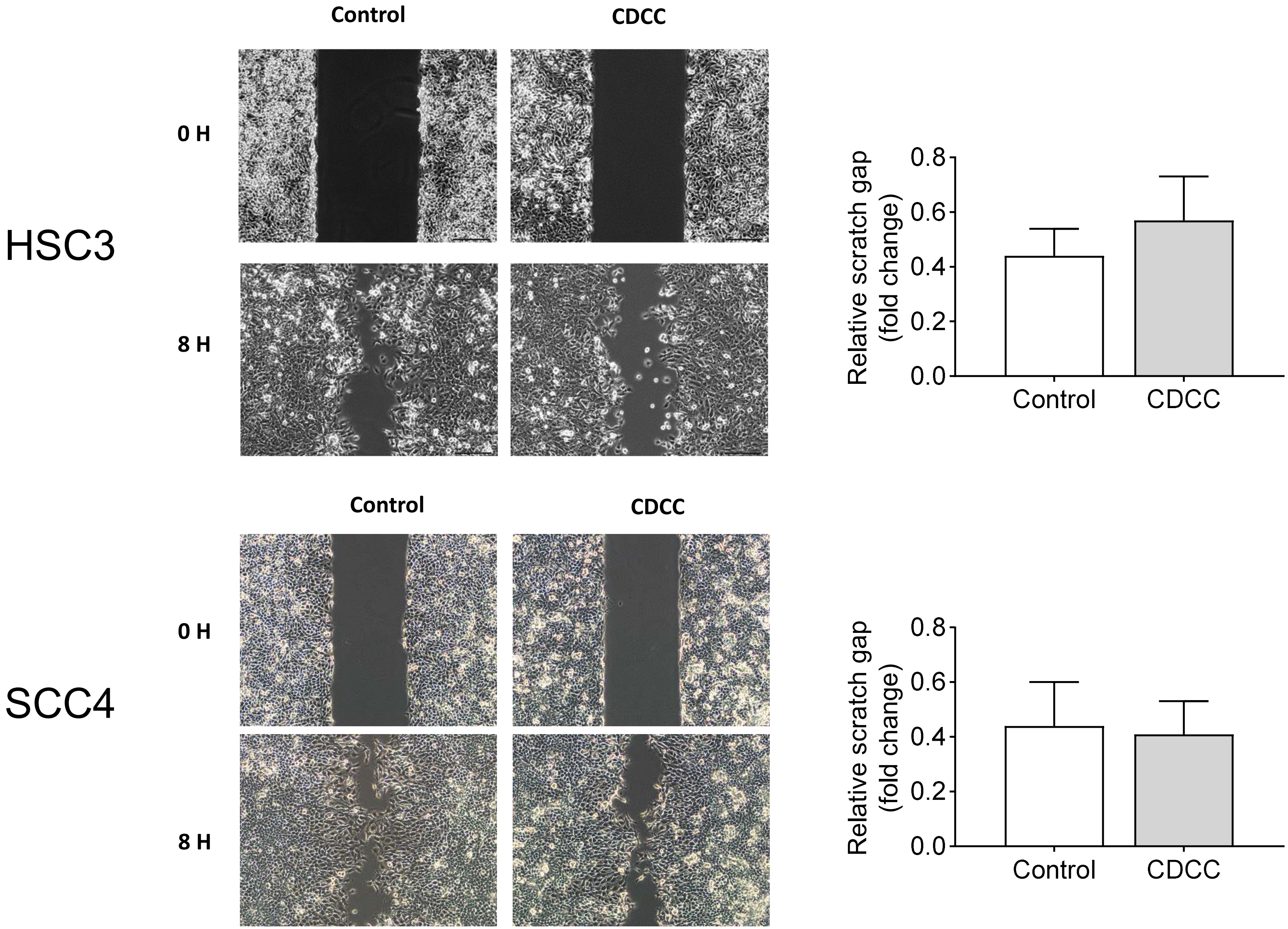
HSC3
SCC4
Control
CDCC
0 H
8 H
Relative scratch gap (fold change)
0.0
0.2
0.4
0.6
0.8
Control
CDCC
